# Bio-inspired backpropagation-free training for optical neural networks

**DOI:** 10.1038/s41377-026-02394-3

**Published:** 2026-07-06

**Authors:** Tingxuan Li, Yibo Dong, Kun Tu, Yuchao Zhang, Shu Li, Yuyang Duan, Peng Sun, Min Gu, Jing Wang

**Affiliations:** 1 School of Artificial Intelligence Science and Technology, University of Shanghai for Science and Technology, Shanghai, China; 2 Institute of Photonic Chips, University of Shanghai for Science and Technology, Shanghai, China

**Keywords:** Lithography, Fibre optics and optical communications

## Abstract

Optical Neural Networks (ONNs) can perform matrix operations at the speed of light and are expected to bring revolutionary advances in energy-efficient computing. However, traditional ONNs (T-ONNs) rely heavily on error backpropagation algorithms, which are fundamentally incompatible with physical optical systems due to the lack of reciprocal error paths and extreme sensitivity to fabrication imperfections. In this paper, we propose a bio-inspired backpropagation-free optical neural network (B-ONN) that circumvents gradient backpropagation through a layer-wise target propagation mechanism. By introducing trainable error convolution kernels, each layer can perform local learning based on target signals rather than chain-ruled gradients, eliminating the need for precise optical conjugation between forward and backward paths. Experimental results demonstrate that B-ONN achieves comparable accuracy to T-ONN on both the MNIST and Fashion-MNIST datasets, with 93.25% and 82.28% accuracy, respectively. Moreover, B-ONN demonstrates superior robustness against phase noise (75% accuracy at σ ≈ 0.4π) and alignment errors (75% accuracy within ±3 pixels). We physically validated B-ONN using a programmable spatial light modulator (SLM) system, achieving 95% accuracy in handwritten digit recognition. Furthermore, we successfully implemented chip-scale integration via nano printing, maintaining 94% accuracy, confirming B-ONN’s effective transferability from programmable setups to fixed devices. Crucially, B-ONN learns smooth phase distributions that provide inherent structural robustness without requiring noise-augmented training. Its local learning rules also support asynchronous parallel updates in scalable deep architectures. This work opens a practical and feasible path for deploying optical computing systems in real-world applications.

## Introduction

Artificial intelligence (AI) has fundamentally reshaped scientific and industrial paradigms, yet the exponential growth in model complexity is pushing electronic hardware toward its thermal and bandwidth limits^1–3^. Optical Neural Networks (ONNs), particularly wavefront-engineered architectures like Diffractive Deep Neural Networks (D²NN)^4–6^, offer a compelling solution by performing massive parallel computations at the speed of light with minimal energy consumption. However, the widespread deployment of ONNs is severely hindered by training inefficiencies. The standard backpropagation (BP) algorithm relies on global error chains and precise gradient calculations, necessitating strict physical symmetry between forward and backward optical paths^7–9^. Implementing this symmetry is arduous due to hardware misalignments, aberrations, and the non-reciprocal nature of many optical components^8^. Moreover, in emerging pre-sensor computing, black-box optical transmission leads to a discrepancy between BP-based gradients and hardware stochasticity, making this global optimization inaccessible^10,11^.

To bridge the gap between rigid physical modeling and scalable learning, backpropagation-free algorithms have emerged, shifting the focus toward localized hardware-efficient learning. Early Direct Feedback Alignment (DFA) schemes bypassed the requirement for reciprocal weight symmetry by simplifying updates into local scalar products, significantly reducing data movement in photonic co-processors^12–14^. Yet, this random projection approach struggles to align with complex spatial features in convolutional architectures^12^, and its reliance on digital forward inference restricts end-to-end optical efficiency^14^. Beyond random feedback, another pathway leverages contrastive learning, most notably the Forward-Forward Algorithm (FFA), which replaces conventional backpropagation with layer-wise local objective functions^10,15^. FFA enables in-situ updates without an explicit backward model by maximizing internal performance metrics through dual forward passes involving both positive data and negative data. Nevertheless, since this paradigm relies on a layer-wide scalar objective rather than high-dimensional vector gradients, it deprives high-resolution diffractive layers of pixel-level directional guidance, often leading to suboptimal convergence in systems with massive degrees of freedom. Seeking to reconcile robust physics with directional precision, the Physical Local Learning (PhyLL) framework computes local updates by evaluating the cosine similarity between differential forward passes^16^. While remarkably universal and robust to unknown physics, PhyLL requires detailed and sequential characterizations of individual layer behaviors, inherently restricting its guaranteed high-dimensional scaling for real-time, large-scale applications^17^. Concurrently, efforts leveraging physical reciprocity^18–20^ have achieved model-equivalent accuracy, though they are typically restricted to spatially symmetrical reciprocal systems. Although these diverse methodologies have significantly advanced the field, they collectively highlight a persistent trade-off between physical hardware expressivity and algorithmic learning efficiency. As such, the realization of a paradigm that enables architectural expansion and vector steering remains a critical yet elusive objective for large-scale optical neural networks.

Inspired by local error-driven synaptic plasticity in biological circuits^21–25^ (Figs. S1–2), we introduce the Bio-inspired Optical Neural Network (B-ONN), a backpropagation-free framework that utilizes layer-wise target propagation and trainable error convolution kernels to enable independent local learning^26–29^. Obviating the rigid necessity for precise optical reciprocity, B-ONN achieves classification performance on MNIST and Fashion-MNIST comparable to traditional backpropagation-based ONNs (gap < 0.5%) while ensuring superior convergence in deep architectures. Our experimental validations—conducted across both programmable spatial light modulator systems and chip-scale integration—demonstrate high-fidelity execution with accuracies of 95% and 94%, respectively. Notably, B-ONN naturally evolves smooth, physically realizable phase profiles that confer inherent robustness against phase noise^30–33^ and misalignments^25^ without necessitating noise-aware training. These results establish B-ONN as a scalable, hardware-compatible paradigm that bridges biological adaptability with optical computing, marking a significant stride toward robust, energy-efficient, and fabrication-ready intelligent photonic systems.

## Results

### Design principle and underlying physics of B-ONN

**Fig. 1:**
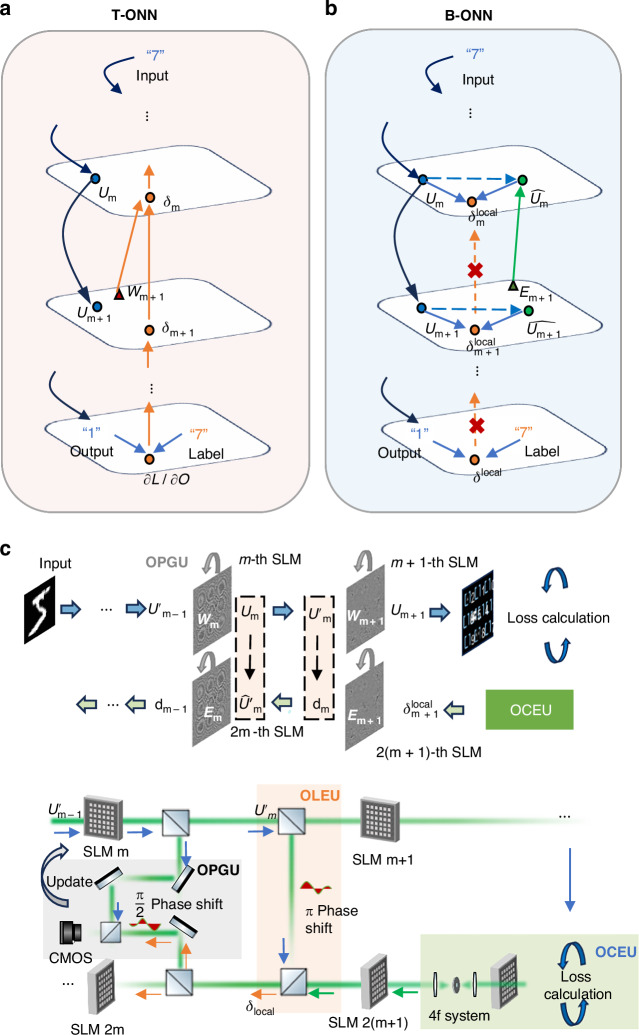
Framework of the B-ONN. **a**, **b** Schematic comparison of T-ONN and B-ONN. T-ONN relies on chain rule-based error backpropagation (orange arrows) for weight updates. B-ONN architecture uses locally generated errors ${\delta }_{m}^{{local}}$ and a trainable error kernel ${E}_{m+1}$ (green) for layer-wise target propagation. **c** All-optical in-situ training implementation of B-ONN. The physical implementation of our system integrates three dedicated optical units that directly map the core algorithmic operations: the OCEU, implemented using phase-only spatial light modulators (SLMs), encodes both the convolutional layers and error kernels; the OLEU structured as a Mach–Zehnder interferometer (MZI), performs optical subtraction for local error computation; and the OPGU is physically realized at the detection port (BS + CMOS). The system performs phase-shifting interferometry at this interface, where the SLM or a phase modulator introduces sequential phase steps to extract the complex gradient from the interference intensity patterns

The physical implementation of B-ONN is centered on partitioning global optimization into decentralized, wavefront-driven update rules (Fig. 1a, b). To operationalize this scheme, the training cycle is structured into two interlinked optical phases. In the forward path, each diffractive layer modulates the incident wavefront using a trainable phase mask to perform spatial feature encoding (see text S1 for details). Complementing this forward transformation, we introduce a learnable error convolution kernel *E* at each layer (Fig. 1b). During training, *E* is optimized to self-consistently approximate the inverse of the forward diffractive operator, thereby translating high-dimensional error signals into spatially-resolved local targets (see text S2). B-ONN resolves these targets through a monolithic optical pipeline to perform gradient computation entirely in the optical domain, which in turn eliminates the latency and energy bottlenecks of electronic backpropagation (Fig. 1c).

As illustrated Fig. 1, the network architecture is defined as a $m$-layer (*m* = 1, 2, 3..., *k*, *k* + 1) system, where the first $k$ layers are phase modulation layers of the optical neural network, and the *k* + 1-th layer is the detection layer. For the $m$-th (*m* = 1, 2, 3..., *k*-1) convolutional layer, the optical field propagates via diffraction and is modulated by a trainable random phase mask for feature extraction^25,34^ (Fig. S3). A key step in B-ONN is the definition of a local error signal ${\delta }_{m}^{{local}}$ for each layer, which is computed directly from the layer’s desired output $\widehat{{U}_{m}^{{l}_{m}}}$ and its actual output ${U}_{m}^{{l}_{m}}$, eliminating the need for signal propagation from subsequent layers: ${\delta }_{m}^{{local}}={U}_{m}^{{l}_{m}}-\widehat{{U}_{m}^{{l}_{m}}}$. The desired output (target) $\widehat{{U}_{m}^{{l}_{m}}}$ is generated through a target propagation mechanism (see text S3 for more details) that integrates the forward output with error feedback: $\widehat{{U}_{m}^{{l}_{m}}}={U}_{m}^{{l}_{m}}-\mathrm{F}(\beta {E}_{m+1}^{{l}_{m+1}})\bigotimes {\delta }_{m+1}^{{local}}$. As presented in text S4, $\beta \,\in \,[\mathrm{0,1}]$ is a scaling factor (Fig. S4), and ${E}_{m+1}^{{l}_{m+1}}=\exp (i{\theta }_{m+1})$ represents the trainable error convolution kernel, which is the core innovation of our method. In contrast to the transposed weight matrix requiring precise conjugation in traditional backpropagation, ${E}_{m+1}^{{l}_{m+1}}$ is an independently learnable parameter matrix that adaptively learns to approximate the inverse transformation during training (Figs. S5–7). Its update rule is given by: Δ*θ*_*m*_ = *γ*Δϕ_*m*_, where *γ*∈[0.01, 0.1] is the learning rate for the error phase, and Δϕ_*m*_ is the gradient of the forward stochastic phase^35^. This design allows the training objective of the error kernel to approach, rather than strictly equal, the inverse of the forward parameters ${R}_{m}^{{l}_{m}}$. Consequently, the stringent requirement for optical time-reversal symmetry is eliminated, enabling the network to be compatible with non-reciprocal optical devices^12^.

### All-optical in-situ training implementation

To validate the physical feasibility of the proposed training framework, we designed a complete all-optical in-situ training system (Fig. 1c, text S5). This implementation physically maps the aforementioned algorithm onto optics through three key components:

1. Optical Complex-Amplitude Encoding Unit (OCEU): We developed an Optical Complex-Amplitude Encoding Unit (OCEU) to enable complex-field modulation using a single phase-only spatial light modulator (SLM). The OCEU operates on a phase-carrier encoding principle, decomposing a complex optical field ${E}_{m+1}^{{l}_{m+1}}=\exp (i{\theta }_{m+1})$ into two complementary phase-only components that are holographically synthesized on the SLM. This unit provides the critical hardware foundation for generating complex error signals entirely in the optical domain.
2. Optical Local Error Unit (OLEU): To compute the local error signal ${\delta }_{m}^{{local}}$, which requires an optical subtraction operation, we constructed an Optical Local Error Unit (OLEU) based on a Mach-Zehnder interferometer. By introducing a $\pi$-phase shift in the reference arm, the OLEU performs destructive interference to achieve real-time subtraction directly in the optical domain. This unit avoids the information loss associated with photoelectric conversion, enabling the generation of local error signals for subsequent in-situ training steps.
3. Optical Parallel Gradient Unit (OPGU): The computation of gradients $\Delta {\phi }_{m}=2{\mathrm{Re}}\left\{i\cdot F\left({P}_{m}\right)\cdot {U}_{m}^{{l}_{m}}\left({-r}_{m}\right)\odot \,{\delta }_{m}^{{local}}\right\}$ is implemented in parallel through spatial interference. Our Optical Parallel Gradient Unit (OPGU) allows the local error optical field and the phase modulation term to interfere synchronously across the entire spatial plane. A single exposure of a CMOS is then sufficient to capture the gradient information for all pixels. This OPGU framework enables massive parallel computation, improving computational efficiency by several orders of magnitude.

### B-ONN for image classification

**Fig. 2:**
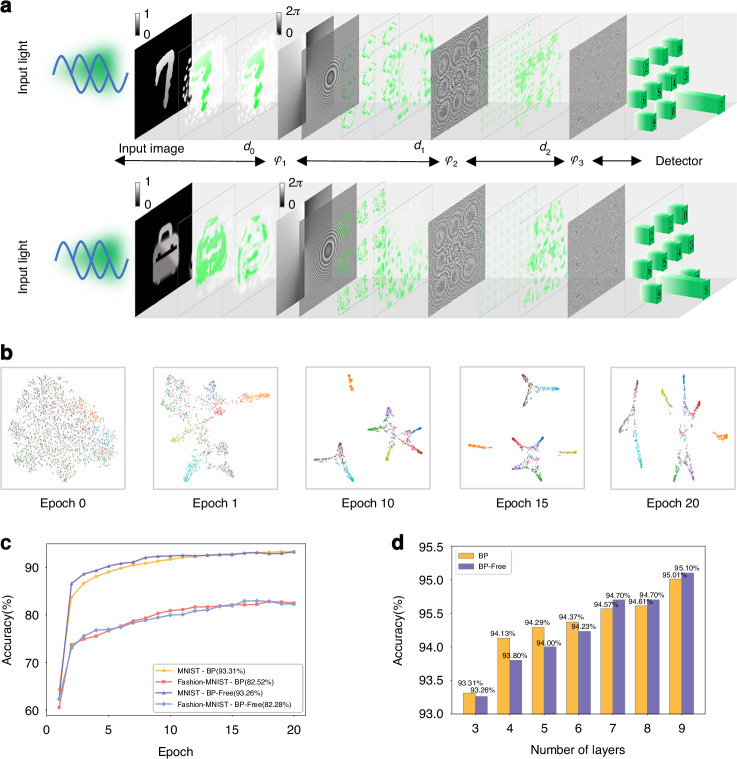
Performance of our B-ONN on MNIST and Fashion-MNIST datasets. **a** Schematic of the B-ONN for image classification. An input image is processed through consecutive optical layers, each comprising a free-space propagation distance$\,d$ and a phase modulation ϕ, before being detected. **b** t-SNE visualization (perplexity=30) of the feature space at the final layer of the B-ONN on the MNIST dataset, shown at different training epochs (0, 1, 10, 15, 20). **c** Test accuracy versus training epochs for the three-layer B-ONN and T-ONN on both datasets. **d** Comparison of the final test accuracy between the B-ONN and the T-ONN across different network depths (3 to 9 layers)

The classification efficacy of B-ONN was first evaluated on MNIST and Fashion-MNIST datasets, benchmarked against traditional optical convolutional neural networks (T-ONN) (Fig. 2a, text S6). The feature extraction layer employed nine convolutional kernels, and the cross-entropy loss function was used throughout training. It is worth noting that in the B-ONN model, hyperparameters *β* and *γ* primarily influence convergence speed rather than final accuracy. Through systematic parameter optimization, we set *β* = *0.9* and *γ* = *0.1* to achieve favorable convergence performance. Its feature-learning capability is qualitatively captured in Fig. 2b, where t-SNE visualization demonstrates clear class separation within the representation space of the final layer. Quantitatively, B-ONN achieves near-parity with T-ONN in a three-layer setup, yielding a marginal accuracy gap of less than 0.5% (Fig. 2c). Validation over an extended training duration further confirms B-ONN’s superior convergence stability while maintaining this performance ceiling (Fig. S8).

Beyond basic benchmarks, the architectural scalability of B-ONN was examined by systematically varying the network depth from 3 to 9 layers. While T-ONN maintained a marginal lead in shallower configurations (3–6 layers), a performance crossover occurred at 7 layers, where B-ONN began to outperform T-ONN, ultimately reaching a peak accuracy of 95.10% in the 9-layer configuration (Fig. 2d). We attribute this depth-dependent performance crossover to the fundamental trade-off between the precision of global optimization and the stability of error propagation. In shallower networks (3–6 layers), T-ONN benefits from the exactness of the chain-rule-based gradients, which can effectively fine-tune parameters without significant signal attenuation. However, as depth increases (7–9 layers), T-ONN encounters gradient instability (vanishing/exploding gradients) typical of deep physical systems (see Fig. S5). In contrast, B-ONN employs a local target propagation mechanism that decouples the layers, maintaining stable gradient distributions regardless of depth. These results indicate that B-ONN not only matches the performance of traditional backpropagation-based approaches across varying depths but also exhibits superior scalability in deeper networks, offering important technical support for the practical deployment of optical neural networks.

### Architecture-inherent noise robustness of B-ONN

The resilience of B-ONN against real-world environmental perturbations was scrutinized through a series of systematic noise tolerance tests. Models were initially optimized under idealized, noise-free conditions to simulate a controlled laboratory environment before being subjected to defective testing conditions. This mimics practical deployment scenarios, where a model optimized under ideal laboratory conditions must cope with inevitable physical imperfections introduced during optical system fabrication and alignment.

**Fig. 3:**
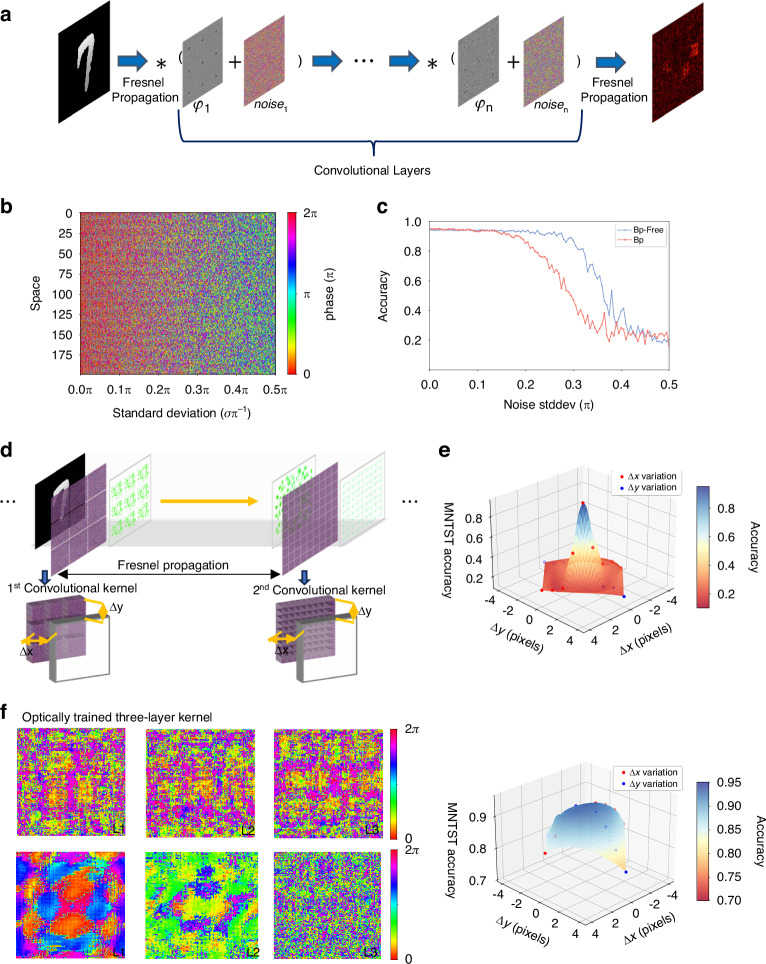
Robustness evaluation of B-ONN against system noise and alignment error. **a** Schematic of the robustness testing pipeline. A digitally propagated image is sequentially superimposed with noisy phase masks *φ*_1_ to *φ*_*n*_ and standard convolutional kernels, with the final output obtained through multiple Fresnel propagations. **b** Representative phase noise patterns with increasing standard deviation (*σ*). **c** Classification accuracy of the B-ONN and T-ONN models under different levels of phase noise (*σ*). **d** Schematic diagram of network alignment errors along the x- and y-axis (Δ*x*, Δ*y*). **e** Impact of lateral misalignment (Δ*x*, Δ*y*) on the classification accuracy for the T-ONN (top) and B-ONN model(bottom). **f** Phase distributions of the convolutional kernels learned by the T-ONN model (top) and the B-ONN model (bottom)

We first evaluated the model’s tolerance to phase perturbations to simulate hardware-intrinsic non-idealities (Fig. 3a). Additive Gaussian phase noise with mean *μ* = 0 and standard deviation σ ranging from 0 to 0.5π was applied (Fig. 3b). Physically, this stochastic noise models the surface roughness and etching depth variations inherent in micro-nano fabrication (e.g., TPP processes), as well as the phase quantization errors and electronic fluctuations typical of spatial light modulators (SLMs). As shown in Fig. 3c, both models achieved over 90% accuracy under noise-free conditions. However, as noise increased, T-ONN performance degraded sharply (showing noticeable fluctuations beyond *σ* > 0.2π), whereas B-ONN exhibited remarkable stability, maintaining approximately 75% accuracy even under strong noise (*σ* ≈ 0.4π). This robustness stems from B-ONN’s target propagation optimization mechanism. By employing trainable error convolution kernels that guide the network toward convergence in flat regions of the loss landscape, B-ONN learns smooth phase profiles with gentle gradients. These profiles inherently suppress high-frequency disturbances in the spectral domain, functioning as a built-in low-pass filter.

We further tested robustness against lateral misalignment of diffractive elements to emulate environmental and assembly constraints (Fig. 3d, e). This misalignment physically corresponds to mechanical positioning errors during system integration and potential optical axis drifts caused by environmental vibrations or thermal expansion. B-ONN again demonstrated superior tolerance. Within a misalignment range of ±3 pixels, B-ONN maintained accuracy above 75%, while T-ONN suffered a sharp drop from 90% to below 60% with only 1–2 pixels of shift. The smooth and delocalized phase profiles learned by B-ONN yield more continuous optical responses in the spatial domain, accommodating approximately 2–3 times larger alignment errors than conventional approaches.

This generalized robustness can be attributed to fundamental differences in the optical transfer functions learned by B-ONN compared to T-ONN. As illustrated in Fig. 3f, T-ONN’s phase profile (top) exhibits high-frequency, localized sharp structures. Although such patterns enable high-precision control under ideal conditions, they are highly sensitive to perturbations. In contrast, B-ONN’s phase map (bottom) is globally smooth with gradual transitions, endowing the system with greater environmental adaptability. From an optimization perspective, B-ONN converges to wide, flat minima rather than narrow local optima, resulting in strong inherent noise resistance—even in the absence of explicit noise-aware training (see more details in text S7). This property is of significant practical value for deploying high-tolerance optical systems under non-ideal conditions.

### Experimental demonstration of B-ONN

**Fig. 4:**
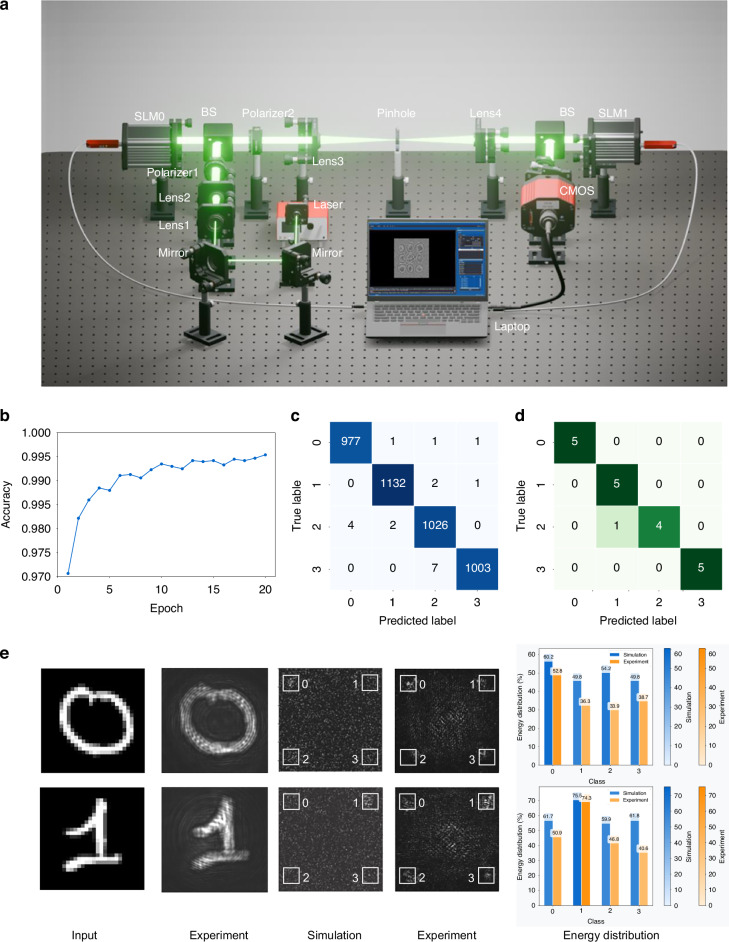
Simulation and experimental results of B-ONN for handwritten digits classification. **a** Optical experimental setup. The system consists of a laser source, an amplitude-type SLM (SLM0) for input encoding, lenses (L1-L4) for beam expansion and scaling, a phase-type SLM (SLM1) for phase modulation, and a CMOS camera for output detection. **b** testing accuracy of the network over epochs on the handwritten digit dataset (classes 0–3). **c** Confusion matrix of the classification results obtained from numerical simulation using 4,157 test samples. **d** Confusion matrix of the classification results obtained from the physical optical experiment using 20 test samples. **e** Comparison of simulation and experimental results for two representative input digits (‘0’ and ‘1’)

Building upon theoretical validation, we experimentally demonstrated the proposed B-ONN architecture using a programmable spatial light modulator (SLM) system (Fig. 4a) to perform a four-class handwritten digit recognition task (digits 0, 1, 2, 3). Detailed hardware specifications and optical components are provided in the Materials and Methods section. The optical system employs a 532 nm laser source expanded via a 4 f relay system. The input data is encoded onto an amplitude-modulated SLM (SLM0), and the beam subsequently propagates through a second 4 f relay system. This intermediate stage serves to demagnify the wavefront (0.75×) to precisely match the pixel dimensions of the downstream phase-modulated SLM (SLM1), which implements the trained phase weights. The modulated optical field is finally captured by a CMOS camera for data processing via a laptop.

The network converged within only 5 training epochs, achieving a test accuracy of 99.5% in simulation (Fig. 4b). Out of the test set, 4157 samples were used for numerical validation, and 20 representative samples were physically tested. The confusion matrices in Fig. 4c (simulation) and Fig. 4d (experiment) summarize the classification results, with the physical system attaining 95% accuracy. Figure 4e details the captured output intensity distributions for representative input digits. Quantitative analysis of the energy distribution histograms (right panels) confirms that the B-ONN effectively functions as a layout-router, with the majority of optical energy accumulated in the specific detector sub-regions corresponding to the ground truth labels. Comparing the two, the numerical simulations exhibit a more dispersed intensity distribution, revealing detailed high-order diffraction patterns and sidelobes. In contrast, the experimental results appear more concentrated within the target region with a cleaner background. This phenomenon is physically attributed to the limited numerical aperture (NA) of the optical relay system, which acts as a spatial low-pass filter suppressing high-frequency diffraction components, combined with the sensitivity threshold of the CMOS camera, which naturally filters out the low-intensity sidelobes present in the simulation.

### Chip-scale integration of B-ONN via diffractive architectures

**Fig. 5:**
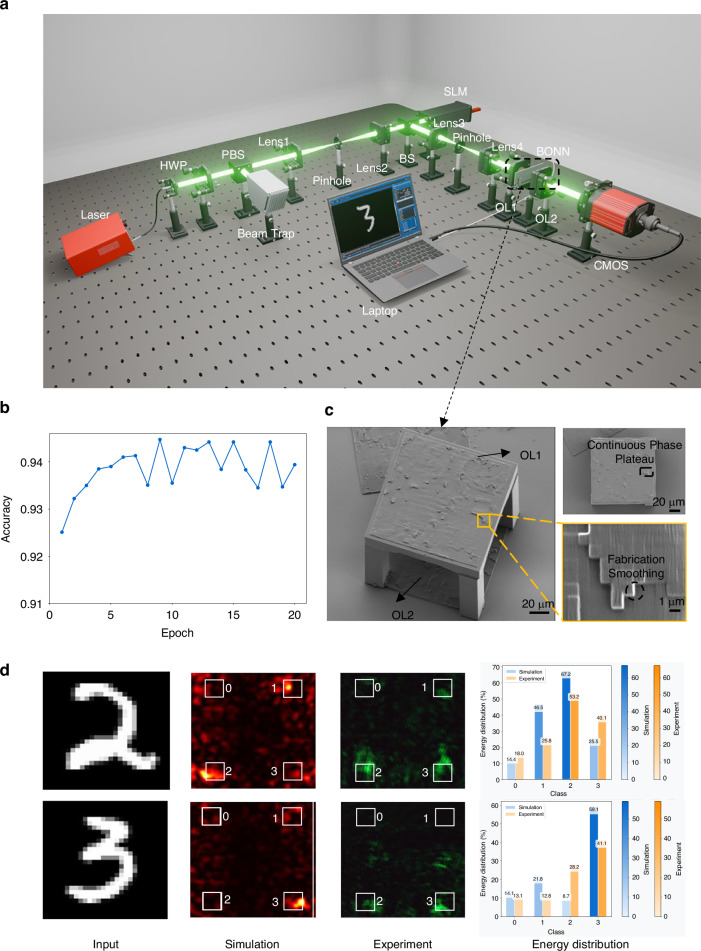
Simulation and experimental results of 2-layer chip with our B-ONN for handwritten digits classification. **a** Schematic of the two-layer B-ONN optical setup, the system consists of two diffractive layers (OL1 and OL2 representing optical layer 1, layer 2 respectively). **b** Testing accuracy of the two-layer B-ONN over epochs on the handwritten digit dataset (classes 0–3). **c** Scanning electron microscope (SEM) image of the fabricated two-layer B-ONN device. Key structural components corresponding to the optical layout in **a** are labeled, including the ‘OL1’ and ‘OL2’. The insets provide high-magnification views of the diffractive structures, where ‘Continuous Phase Plateau’ marks the flat surface areas of the phase blocks, and ‘Fabrication Smoothing’ indicates the transition edges between adjacent phase steps. **d** Comparison of simulation and experimental results for representative input digits

Advancing from reconfigurable SLM platforms toward miniaturized photonic solutions, we extended its application to a chip-scale integrated diffractive deep neural network (D²NN) (Figs. S9–10) architecture (Fig. 5a and text S8). The model was trained on the same four-class subset of the MNIST handwritten digit dataset. As shown in Fig. 5b, the B-ONN-driven optimization exhibited convergence behavior similar to that of the OCNN, reaching stability after only 5 epochs and achieving 94% accuracy on the validation set after 20 epochs. Figure 5c presents a scanning electron microscopy (SEM) image of the physically fabricated device. A comparison between simulation results and experimental measurements (Fig. 5d) reveals strong agreement in the spatial routing of optical signals, as corroborated by the energy percentage bar charts. Specifically, for inputs ‘2’ and ‘3’, the measured energy is predominantly concentrated in the correct target quadrants. A notable difference is that the simulation results display highly concentrated energy peaks accompanied by diffractive interference patterns in the background, whereas the experimental spots appear more diffuse and expanded, overflowing the target boundaries. This physical broadening stems from the continuous surface profile of the TPP-fabricated structure. As explicitly visualized in the high-magnification SEM insets of Fig. 5c, the regions annotated as ‘Fabrication Smoothing’ reveal that the TPP polymerization process transforms the theoretically sharp step edges into rounded, continuous slopes.

Crucially, this fabrication characteristic aligns perfectly with the algorithmic properties of B-ONN. Unlike traditional methods that may demand sharp, fragmented phase variations, B-ONN inherently learns spatially continuous phase weights (marked as ‘Continuous Phase Plateau’ in Fig. 5c). This smoothness implies that our training method acts as a natural low-pass filter, creating a specialized synergy where the algorithm anticipates and adapts to the physical constraints of the manufacturing process. Consequently, the fabricated device maintains high performance despite the manufacturing smoothing, confirming that B-ONN is not only a theoretical model but a fabrication-ready solution for integrated photonics.

## Discussion

This study introduces a backpropagation-free training framework for optical neural networks, effectively addressing the incompatibility between global gradient-based learning and the physical constraints of optical systems. Unlike traditional schemes where digital optimizers bear the entire computational burden, B-ONN utilizes optical interference to execute complex spatial transformations at the speed of light. While standard digital optimizers like Adam are employed, they serve primarily to accelerate numerical convergence and stabilize temporal dynamics. This synergy ensures intrinsically smooth and noise-resilient phase profiles, faithfully emulating the neuro-computational principle of learning rules^36–38^.

It must be noted that the performance gains of B-ONN stem from a strategic space-for-intelligence trade-off. B-ONN incurs approximately 3 times the physical space overhead to achieve an 18-fold reduction in dynamic electrical memory traffic for a 9-layer architecture (text S9, S10). Similarly, the system expands its total physical footprint through the cascading of multiple independent OPGU and OLEU modules to realize the simplification of electrical primitives into low-power subtractions. While these trade-offs effectively bypass the memory wall, they shift the systemic burden toward increased hardware complexity and integration precision. As a result, the future development of monolithic photonic integration will be essential to compress these physical dimensions and enhance the structural robustness for large-scale B-ONN implementations.

As optical computing transitions from laboratory research toward large-scale practical applications, training methodologies must evolve to synchronize with the advancements in photonic integration. Future work will focus on exploring lightweight target generation mechanisms, such as low-rank approximation^39^ or random projection^40^, to further compress the physical pixel footprint to ease the spatial requirements for on-chip density^41^. Simultaneously, the pursuit of monolithic photonic integration^42^ and advanced 3D heterogeneous packaging^43^ will be paramount, aiming to consolidate the cascaded optical modules into a more compact unit, mitigating the current systemic burden of hardware complexity.

## Materials and methods

### Network architecture and forward propagation

We define the network to consist of $m$ layers, where the first $k-1$ layers are convolutional layers, the $k$-th layer is a fully connected output layer, and the $k+1$-th layer is the detection layer.

For the *m*-th convolutional layer$\,(m\mathrm{=}1\mathrm{,}2\mathrm{,}3\mathrm{,...,}k-1)$, the optical field propagation is described as:

1 $${U}_{m}^{{lm}}({r}_{m})={P}_{m}({r}_{m})\cdot {U}_{m-1}^{{l}_{m-1}}(-{r}_{m})\otimes \bar{F}({R}_{m}^{{l}_{m}})$$

where: $\mathrm{m}$ is the layer index, ${l}_{m}$ is a multi-dimensional index identifying which feature map from the previous layer serves as input, ${r}_{m}=({u}_{m},{v}_{m})$ denotes the spatial coordinates in the $\mathrm{m}$-th layer plane, ${P}_{m}$ is the propagation phase factor accounting for the phase modulation from layer $m-1$ to $\mathrm{m}$.

For *m* = 1, *P*_*m*_ = 1 indicates direct imaging from the input (layer 0) to the first layer with no additional phase.

For *m* ≥ 2, $${P}_{m}=\exp \left[{ik}/\left(2f\right)\left({u}_{m}^{2}+{v}_{m}^{2}\right)\right]$$

${U}_{\left(m-1\right)}^{\left({l}_{\left(m-1\right)}\right)}\left(-{r}_{m}\right)$ represents the feature optical field from the $m-1$-th layer, where the coordinate inversion reflects the image-reversal property of the imaging system. ${R}_{m}^{\left({l}_{m}\right)}$ denotes the trainable random phase mask applied at the m-th layer for phase modulation, with ${R}_{m}^{\left({l}_{m}\right)}=\exp \left(i{\varPhi }_{m}\right)$.

The symbol $\otimes$ denotes convolution, implemented via an optical 4 f system. $\mathrm{F}$ represents the Fourier transform, and $\bar{\mathrm{F}}$ denotes the inverse Fourier transform.

For the $k$-th fully connected output layer:

2 $${U}_{k}^{\left({l}_{k}\right)}\left({r}_{k}\right)=\left({U}_{\left(k-1\right)}^{\left({l}_{\left(k-1\right)}\right)}\cdot {R}_{k}^{\left({l}_{k}\right)}\right){vec}* {H}_{k}^{\left({l}_{k}\right)}$$

where: ${vec}$ denotes vectorization of the optical field distribution, represents matrix multiplication, ${H}_{k}^{\left({l}_{k}\right)}$ is the Fresnel diffraction weight matrix corresponding to the feature map.

The intensity at the detection layer (output plane) is given by:

3 $$O({r}_{k+1})=|{U}_{k}{|}^{2}={\left|\mathop{\sum }\limits_{{l}_{k-1}}{U}_{k}^{{l}_{k}}\left({r}_{k}\right)\right|}^{2}$$

Here, $O\left({r}_{\left(k+1\right)}\right)$ describes the optical intensity distribution at the output plane of the fully connected layer, i.e., the detection layer of the network.

### Backpropagation-free training algorithm

We define the loss function as $L\left(O,T\right)$, where $O$ represents the output optical field intensity and $T$ denotes the ground truth label.

For multi-class classification problems, the cross-entropy loss function is adopted. The error optical field at the final layer is computed as:

4 $${\delta }_{k+1}=\frac{\partial L}{\partial O}\odot {U}_{k}^{* }$$

where: ⊙ denotes element-wise multiplication, ${U}_{k}^{* }$ is the complex conjugate of the output optical field from the $k-t\mathrm{h}$ layer. The conjugation ensures correct computation of the error optical field.

For any intermediate layer $m$ where $m\ne k+1$, the gradient is calculated as:

5 $${\Delta \phi }_{m}=2Re\left\{{iF}\left({P}_{m}\right)\cdot {U}_{m}^{{l}_{m}}\left(-{r}_{m}\right)\odot {\delta }_{m}^{{local}}\right\}$$

where: ∆ϕ_m_ is the gradient of the modulated random phase ϕ_m_, ${\delta }_{m}^{{local}}$ is the local error at the $m$-th layer, computed independently per layer without inter-layer propagation, $\mathrm{Re}$ denotes the real part.

The local error is defined as:

6 $${\delta }_{m}^{{local}}={U}_{m}^{{l}_{m}}-\widehat{{U}_{m}^{{l}_{m}}}$$

Mathematically, this physical interference pattern is equivalent to the gradient of a local proxy loss function ${\mathrm{L}}_{m}=\frac{1}{2}\Vert {U}_{m}-{\hat{U}}_{m}{\Vert }_{2}^{2}$ with respect to the layer’s output (${\delta }_{m}^{{local}}={\nabla }_{{U}_{m}}{\mathrm{L}}_{m}$). Therefore, by physically minimizing this local error signal via phase modulation, the layer performs exact gradient descent on its local objective, independent of the instantaneous state of the rest of the network. Where ${\hat{U}}_{m}$ is the target optical field.

The target optical field is constructed as:

7 $$\widehat{{U}_{m}^{{l}_{m}}}={U}_{m}^{{l}_{m}}-F\left({\beta E}_{m+1}^{{l}_{m+1}}\right)\bigotimes {\delta }_{m+1}^{{local}}$$

where: *β* is a scaling factor controlling the contribution of the error optical field to the target, $F$ denotes Fresnel diffraction applied to the error optical field.

Notably, ${E}_{m+1}^{{l}_{m+1}}=\exp (i{\theta }_{m})$ is a trainable error phase applied at the $m+1$*-th* layer. It is an independent, trainable parameter matrix—not a transpose or fixed transformation of ${R}_{m+1}^{{l}_{m+1}}$. The training objective for ${E}_{m+1}^{{l}_{m+1}}$ is to make its behavior approximate that of the forward parameter ${R}_{m+1}^{{l}_{m+1}}$, though not identically. We define its gradient relative to the forward random phase as:

8 $${\Delta \theta }_{m+1}={\gamma \Delta \phi }_{m+1}$$

Where $\gamma$, typically set within $\left[0.01\mathrm{,}0.1\right]$, serves as the learning rate for updating the error phase.

### Geometric analysis of error kernel mechanism

To quantitatively investigate the operational mechanism of the error convolution kernel $E$ and verify its theoretical role as an approximate inverse operator, we monitored the geometric alignment between $E$ and the forward kernel $R$ throughout the training process. Specifically, we computed the geometric angle ϑ between the flattened weight vector of the learned error kernel, denoted as *v*_*E*_, and the flattened vector of the theoretical pseudo-inverse of the forward kernel, denoted as *v*_R−1_. The angle is defined as:

$\vartheta =\arccos(\frac{{v}_{E}\cdot {v}_{{R}^{-1}}}{|{v}_{E}|\cdot |{v}_{{R}^{-1}}|})$. Ass visualized in Fig. S7, the angle starts at 90°, indicating that the random initialization is orthogonal to the physics of the forward model. As training progresses, this angle rapidly converges to approximately 34°. This specific convergence angle suggests that $E$ does not strictly become the mathematical inverse (which would correspond to 0°), but rather acts as a learned regularized inverter. It aligns significantly with *R*^−1^ to effectively deconvolve the error signal while maintaining a necessary deviation to ensure numerical stability and robustness against high-frequency noise inherent in the optical system.

### Training dataset processing

Experimental data were sourced from the MNIST and Fashion-MNIST datasets, containing images of handwritten digits and fashion items, respectively. All images were upsampled from an original resolution of 28 × 28 to 84 × 84, and subsequently zero-padded to a uniform size of 112 × 112 pixels. The dataset comprises 60,000 training samples and 10,000 validation samples. In accordance with common practices in the machine learning field, 30% of the training set (18,000 images) was reserved as an independent test set, which did not participate in model training. All images were processed and maintained in grayscale format.

### Sample nanoprinting

The sample fabrication was performed using a commercial two-photon lithography system (Photonic Professional GT, Nanoscribe GmbH), equipped with a femtosecond laser source operating at a central wavelength of 780 nm and a repetition rate of 80 MHz. In dip-in laser lithography mode, a backpropagation-free double-layer diffractive neural network structure was fabricated using IP-Dip photoresist and a high-numerical-aperture oil-immersion objective (Zeiss Plan-Apochromat 63×/1.40 Oil DIC). The fabrication followed a layer-by-layer strategy. First, the bottom support pillars were fabricated with a vertical slice spacing of 0.5 μm and a lateral scanning spacing of 0.2 μm to enhance efficiency. Subsequently, the bottom diffractive layer—serving as the core component for optical field modulation—was fabricated with a vertical slice spacing of 0.213 μm and a lateral scanning spacing of 0.05 μm to ensure structural accuracy. Finally, the connecting pillars and the top diffractive layer were fabricated using the same parameters as the bottom diffractive layer. The optimized process parameters were as follows: the support pillars were fabricated using a laser power of 52 mW and a scanning speed of 20,000 μm s^−1^; the bottom diffractive layer was fabricated with 40 mW and 10,000 μm s^−1^; and the connecting pillars and top diffractive layer employed the same laser parameters as the bottom layer (40 mW, 10,000 μm s^−1^). After printing, the sample was developed in propylene glycol monomethyl ether acetate for 25 min, followed by a 5-min rinse in isopropyl alcohol. To address the structural complexity of the double-layer design, the development time was extended to 40 min to ensure structural integrity. Final drying was achieved via evaporation at room temperature.

### Optical experimental setup

SLM-based Setup (Fig. 4a): The system utilized a continuous-wave solid-state laser operating at 532 nm (MSL-S-532-B, CNI). The beam was expanded and collimated using a 4 f relay system comprising achromatic doublets with focal lengths of f_1_ = 30 mm and f_2_ = 200 mm (Thorlabs). We employed two separate SLMs: an amplitude-modulated SLM (SLM0; FSLM-2K70-A02, Resolution 1920 × 1080, pixel size 8.0 μm, Xi’an Casmicro) for encoding input data, and a phase-modulated SLM (SLM1; FSLM-2K73-P02HR, Resolution 2048 × 2048, pixel size 6.4 μm, Xi’an Casmicro) for implementing the trained phase weights. A second 4 f imaging system with lenses f_3_ = 200 mm and f_4_ = 150 mm (Thorlabs) was interposed between the SLMs to demagnify the wavefront by 0.75×, ensuring precise pixel alignment between the two modulators. The final optical output was captured by a CMOS camera (Thorlabs CS235MU) for data processing.

Chip-scale Setup (Fig. 5a): For the on-chip experiments, a laser source operating at 515 nm was used. The beam was collimated and spatially filtered using a pinhole within a 4 f system to ensure a planar wavefront. The optical path employed a polarizing beam splitter(CCM5-PBS201/M), and a half-wave plate(WPH10M-514) for beam conditioning. The input data was encoded using a reflective Spatial Light Modulator (HOLOEYE LETO-3-CFS-127, HOLOEYE Photonics AG). The modulated beam propagated through the fabricated B-ONN chip structure. The diffracted optical field at the output plane was captured by a CMOS camera (AIC-1201C-USB, JCOPTIX) for subsequent analysis and classification.

## Supplementary information

Supplementary Information for Bio-inspired backpropagation-free training for optical neural networks

## Data Availability

All data needed to evaluate the conclusions in the paper are present in the paper and Supplementary Information. Additional data related to this paper may be requested from the corresponding author, J.W.
