# Supplementary material for: Bio-inspired backpropagation-free training for optical neural networks
Source: Light Sci Appl. 2026 Jul 6;15:305. doi: 10.1038/s41377-026-02394-3 (PMC13338426; doi:10.1038/s41377-026-02394-3)
Supplement: Supplementary file 1 — Supplementary Information for Bio-inspired backpropagation-free training for optical neural networks [file 41377_2026_2394_MOESM1_ESM.pdf]

Supplementary Information for

# Bio-inspired backpropagation-free training for optical neural networks

Tingxuan Li[1,2], Yibo Dong[1,2], Kun Tu[1,2], Yuchao Zhang[1,2*], Shu Li[1,2], Yuyang Duan[1,2], Peng Sun[1,2], Min Gu[1,2*], Jing Wang[1,2*]

[1]School of Artificial Intelligence Science and Technology, University of Shanghai for Science and Technology, Shanghai 200093, China

[2]Institute of Photonic Chips, University of Shanghai for Science and Technology, Shanghai 200093, China

* Corresponding author. Email: wangj@usst.edu.cn, zhangyc@usst.edu.cn, gumin@usst.edu.cn

**Supplementary note 1: Complete Forward Propagation Formulation**

1.1 First Optical Convolutional Layer

The first optical convolutional layer is designed for feature extraction from an input object. The input field distribution is denoted as $I(x_i,y_i)$, where $(x_i,y_i)$ are the coordinates in the input plane $(x,y)$. The layer is positioned in the plane, with the output feature maps located in the plane $(u,v)$. The distances between the input plane, lens plane, and output plane are all $(d_1)$, satisfying the lens law $d_1=2f$, where $f$ is the focal length. The layer comprises four key components integrated into the pupil function:

Lens function: $L(x,y)$, Defined as

$$L(x,y)=P(x,y)\cdot\ exp\left[-i\frac{k}{2f}(x^2+y^2)\right] \tag{S1}$$

which enables optical convolution. The output field, based on Fourier optics, is expressed as

$$U(u,v)=exp\left[ik\frac{u^2+v^2}{2d_1}\right]\left[U_i(-u,-v)\otimes\ h(u,v)\right] \tag{S2}$$

Where $k=2pi/\lambda$, $\otimes$ denotes convolution, and $h(u,v)$ is the point-spread function (PSF), given by the Fourier transform of the pupil function:

$$h(u,v)=F\{P(x,y)\} \tag{S3}$$

Vortex function: $V(x,y)=\exp(il\varphi)$ Enhances edge detection by extracting phase-related features.

Randomly initialized modulation function: $R(x,y)=\exp[iM(x,y)]$ .Where $M(x,y)$ is a trainable phase initialized randomly in $2\pi\cdot[-a,a]$, with $a\in[0.2,0.6]$.

Grating function:

$$G(x,y)=exp\left[i2\pi(f_a x+f_b y)\right] \tag{S4}$$

With spatial frequencies $f_a=f_b=f_1$ , it multiplexes kernels by deflecting light into 9 diffraction directions $(m,n)=\{(0,0),(0,\pm1),(\pm1,0)\}$. The Fourier transform is:

$$F\{G(x,y)\}=\delta\left(f_x-mf_1,f_y-nf_1\right) \tag{S5}$$

leading to shifted outputs in the output plane by $(\Delta u,\Delta v)=(m\Delta_1,n\Delta_1)$ , where $\Delta_1=\lambda f_1 d_1$.

The total pupil function combines these elements:

$$P_1=\sum V_{m,n}R_{m,n}\,G_{m,n} \tag{S6}$$

While the total pupil function $P_1$ (Eq. S6) is mathematically derived from the element-wise product of three phase components, their selection and integration order Vortex $\longrightarrow$ Random $\longrightarrow$ Grating reflects a rigorous logical hierarchy of optical functionality designed for efficient neural computing:

Vortex Phase ( $V$, Feature Inductive Bias): We introduce the vortex phase $exp(il\varphi)$ as the foundational structural prior. It serves as an inductive bias for edge enhancement and orientation selectivity, ensuring that the network is initialized with effective feature extraction capabilities akin to Gabor filters in electronic CNNs.

Random Phase ($R$, Trainable Weight): Superimposed on the vortex structure is the random phase $exp\,[iM(x,y)]$. This component acts as the trainable synaptic weight. By modulating the fixed feature prior defined by $V$, it provides the necessary degrees of freedom for the network to learn task-specific representations through training.

Grating Phase ($G$, Spatial Router): Finally, the grating phase $exp\,[i2\pi(f_a x+f_b y)]$ functions as a spatial carrier. Once the features are extracted ($V$) and weighted ($R$), the grating physically routes the processed signal to designated spatial locations. This enables the spatial multiplexing of multiple convolution kernels onto a single optical plane without mutual interference.

This hierarchical design ensures that a single physical diffractive layer simultaneously performs feature extraction, weight modulation, and parallel channel routing.

The output field is:

$$U_1(u,v)=exp\left[ik\frac{u^2+v^2}{2d_1}\right]\sum_{m,n}U_{m,n}^{(1)}(u\text{-}m\Delta_1,v\text{-}n\Delta_1) \qquad (S7)$$

Where $U_{m,n}^{(1)}=U_i(\text{-}u,\text{-}v)\otimes\ \mathrm{F}\{V_{m,n}R_{m,n}\}$.This generates 9 feature maps tiled in an array.

1.2 Second Optical Convolutional Layer

The second layer processes the output of the first layer, which includes a quadratic phase factor. The input plane is $(u,v)$, and the layer is divided into 3×3 zones corresponding to the first layer's outputs. Each zone has a similar structure to the first layer, with lens function $L_2$ and pupil function $P_2$.

For a zone labeled $(m,n)$, coordinate transformations are applied:

$$u_m^{'}=u\text{-}m\Delta_1,\quad v_n^{'}=v\text{-}n\Delta_1,\quad x_{2,m}^{'}=x_2\text{-}m\Delta_1 \qquad (S8)$$

The output field for the zone is:

$$U_2(u_2,v_2)=Q_{2;m,n}\left[U_1(u,v)\otimes\ h_2(u_2,v_2)\right] \qquad (S9)$$

Where $Q_{2;m,n}$ is a quadratic phase factor, and $\mathrm{h}_2$is the PSF of $P_2$.

The pupil function incorporates a grating function with 9 diffraction directions $(m^{'},n^{'})$:

$$G_{2,m^{'},n^{'}}=exp\left[i2\pi\left(m^{'}f_2x_{2,m}^{'}+n^{'}f_2y_{2,n}^{'}\right)\right] \qquad (S10)$$

leading to shifts $(m^{'}\Delta_2,n^{'}\Delta_2])$ with $\Delta_2=\lambda f_2 d_2$. The output for the zone is:

$$U_{m,n;m^{'},n^{'}}^{(2)}=U_{m,n}^{(1)}\otimes\ F\{V_2R_2\} \qquad (S11)$$

and the total output is:

$$U_2(u_2,v_2)=\sum_{m,n}\sum_{m^{'},n^{'}}U_{m,n;m^{'},n^{'}}^{(2)}(u_2\text{-}m\Delta_1\text{-}m^{'}\Delta_2,v_2\text{-}n\Delta_1\text{-}n^{'}\Delta_2) \qquad (S12)$$

This produces 81 feature maps in an array.

1.3 Third Fully-Connected Layer

The third layer combines the 81 feature maps from the second layer. Each map is labeled by $(m,n;m^{'},n^{'})$, with position in the input plane $(u_2,v_2)$. The output plane is $(u_3,v_3)$, at distance$d_3$.The phase profile includes:

A quadratic phase factor $Q_3=Q_2^{*}$ to cancel previous phase effects.

A randomly initialized modulation function $R_3$.

A grating phase: $G_{3,m,n;m^{'},n^{'}}=exp\left[i2\pi\left(\Delta_{3,m,m^{'}}u_2+\Delta_{3,n,n^{'}}v_2\right)/\lambda d_3\right]$

Where $\Delta_{3,m,m^{'}}=m\Delta_1+m^{'}\Delta_2$, $\Delta_{3,n,n^{'}}=n\Delta_1+n^{'}\Delta_2$.

Using the Huygens-Fresnel principle, the diffraction for a single feature map is:

$$U_{3,m,n;m^{'},n^{'}}=\iint U_2(u_2,v_2)\,G_3R_3Q_3\frac{exp\,(ikr)}{i\lambda d_3}du_2dv_2 \tag{S13}$$

Where $r$ is the distance. In matrix form, this is:

$$\left[U_{3,m,n;m^{'},n^{'}}\right]_{vec}=H^{(3)}_{m,n;m^{'},n^{'}}*[U_2]_{vec} \tag{S14}$$

With $H^{(3)}$as the Fresnel weight matrix. The total output is:

$$U_3(u_3,v_3)=\sum_{m,n}\sum_{m^{'},n^{'}}U_{3,m,n;m^{'},n^{'}} \tag{S15}$$

The final inference result is the intensity $O=|U_3|^2$, detected by a CCD.

1.4 Summary of OCNN Forward Propagation

The forward propagation is summarized by:

First layer convolution producing 9 feature maps.

$$U^{(1)}_{m,n}(u,v)=U_i(-u,-v)\otimes\ F\left(V_{m,n}R_{m,n}\right) \tag{S16}$$

Second layer convolution generating 81 maps.

$$U^{(2)}_{m,n;m^{'},n^{'}}\left(u^{'}_{2,m},v^{'}_{2,n}\right)=exp\left[i\frac{k}{2f}(u^{'2}_{m}+v^{'2}_{n})\right]U^{(1)}_{m,n}(-u^{'}_{m},-v^{'}_{n})\otimes\ F\left(V_{2,m.n;m^{'},n^{'}}R_{2,m,n;m^{'},n^{'}}\right) \tag{S17}$$

Third layer combining maps via Fresnel diffraction.

$$U^{(3)}_{m,n;m^{'},n^{'}}(u_3,v_3)=\left(U^{(2)}_{m,n;m^{'},n^{'}}\cdot\ R_{3,m,n;m^{'},n^{'}}\right)_{vec}*H^{(3)}_{m,n;m^{'},n^{'}} \tag{S18}$$

Output intensity.

$$O(u_3,v_3)=|U_3|^2=\left|\sum_{m,n;m^{'},n^{'}}U^{(3)}_{m,n;m^{'},n^{'}}(u_3,v_3)\right|^2 \tag{S19}$$

**Supplementary note 2: B-ONN Training Procedure**

Pseudo Code of B-ONN Training

Objective Function: $L(O,T)$

Initialization: Number of layers $k+1$, Batch size $B$, Learning rate $\eta$, Trainable phase $\phi_m$ at $m$-th layer , Scaling factor $\alpha, \gamma$, $\Delta\theta_m$ Error phase at $m$-th layer, Local error $\delta_m$ at $m$-th layer.

WHILE NOT CONVERGED

1. Getting a batch of input-output samples from the training dataset
2. Gradient calculation for the batch

FOR $i$ in range (1, B) DO

Forward Propagation

Input: $U_0^i$

For $m$=1 To $k$+1 Do:

$$R_m=exp(i\cdot\phi_m)$$
$$U_m^i=U_{m\text{-}1}^i\odot R_m$$

END FOR

Output: $O^i=U_N^i$

Error Calculation at Output Layer

$$\delta_N^i=\frac{\partial L}{\partial O}\bigg|_{O=O^i}$$

Backward propagation and gradient computation

For $m$=$N$ DOWN TO 1 DO:

Gradient of $\phi_m$:

$$\Delta\phi_m=2Re\left\{i\cdot\ U_m^i\odot R_m^{complex}\odot\delta_m^i\right\}$$

Error propagation to previous layer:

$$E_m^{complex}=exp\left(i\cdot\ \Delta\theta_m\right)$$

$$\widehat{U_{m\text{-}1}^i}=U_{m\text{-}1}^i\text{-}F(\beta\cdot\ E_m^{complex})\odot\delta_m^i$$

$$\delta_{m\text{-}1}^i=U_{m\text{-}1}^i\text{-}\widehat{U_{m\text{-}1}^i}$$

END FOR

END FOR

3. Gradient averaging across the batch

$$\Delta\phi_m=\frac{1}{B}\sum_{i=1}^{B}\Delta\phi_m$$

4. Phase coefficients update

$$\phi_m=\phi_m\text{-}\eta\cdot\ \Delta\phi_m$$
$$\theta_m=\theta_m\text{-}\gamma\cdot\ \Delta\phi_m$$

END WHILE

**Supplementary note 3: Theoretical Foundations of Optical Local Target Propagation**

3.1 The Weight Transport Problem in Optics

In a standard neural network, the update for a weight matrix W at layer m depends on the error $\delta$ from layer $m+1$ propagated through the transpose of the downstream weights $W_{m+1}^{T}$. $\delta_m = \phi'(h_m) \odot (W_{m+1}^{T} \delta_{m+1})$.

In a digital computer, accessing $W_{m+1}^{T}$ is a trivial memory access operation. In a physical optical system, W represents a physical object—a diffractive phase mask or a mesh of interferometers. The "transpose" of this physical object does not exist as a separate entity. To implement rigorous BP, one must physically construct a "backward" optical path that is the exact optical conjugate of the "forward" path. This requires perfect reciprocity, precise alignment, and the ability to modulate light in reverse exactly as it was modulated in forward propagation. Any fabrication imperfection, thermal drift, or misalignment breaks this symmetry, causing the calculated gradient to vanish or explode, thereby destabilizing learning.

3.2 From Feedback Alignment to Target Propagation

To bypass the symmetry requirement, Feedback Alignment (FA) demonstrated that fixed random feedback weights can support learning, provided the alignment angle between the feedback and true gradient is less than 90 degrees. However, FA struggles with deep networks and complex tasks (e.g., ImageNet) as the alignment signal degrades into noise.

The B-ONN adopts the more robust Difference Target Propagation (DTP) framework. DTP decomposes the global optimization into local sub-problems. Instead of propagating gradients, it computes a "virtual target" $\widehat{U}_m$ for each layer. The objective of layer m is to minimize the local reconstruction error:

$$\mathscr{L}_{local} \approx \| U_m - \widehat{U}_m \|^2 \qquad (S20)$$

The critical challenge in DTP is generating a valid target $\widehat{\mathrm{U}}_{\mathrm{m}}$ that effectively guides the layer toward a lower global loss. This requires an approximate inverse mapping of the downstream layers.

3.3 Weight Mirroring via Optical Reciprocity

In a lossless diffractive system, the forward propagation matrix $R$ is unitary. Mathematically, the inverse of a unitary operator is its conjugate transpose ($R^{-1} = R^{\dagger}$). This implies that the ideal backward propagator (the error kernel $E$) should be structurally symmetric to the forward kernel.

To achieve this, B-ONN introduces a symmetry-enforcing update rule :

$$\Delta\theta_E = \gamma\Delta\phi_R \tag{S21}$$

This rule, known as Weight Mirroring, forces the error kernel E to dynamically track the structural evolution of the forward kernel $R$. Unlike standard FA where the forward weights must "chase" a static feedback matrix, Weight Mirroring allows both to evolve in lock-step. By maintaining the structural symmetry $\theta_E \approx \theta_R$, the physical reciprocity of the optical path naturally ensures that E approximates the conjugate transpose $R^{\dagger}$, and thus the inverse $R^{-1}$. This guarantees that the feedback signal maintains a positive projection on the true gradient direction (angle < 90°), ensuring valid descent.

### 3.4 The Gauss-Newton Proxy

The effective learning of the inverse ($E \approx R^{-1}$) provides a profound theoretical advantage: it transforms the optimization from first-order Gradient Descent into an approximation of Gauss-Newton (GN) optimization.

Recent theory establishes that Target Propagation acts as a hybrid between GN and SGD. In the B-ONN, the target is generated via $\widehat{U}_m \approx U_m - \eta E_{m+1}\delta_{m+1}$.

Since $E_{m+1}$ approximates the inverse Jacobian $J^{-1}$ (via the mechanism in Section 3.3), the update direction implicitly incorporates curvature information of the loss landscape :

$$\Delta\phi \propto -J^{-1}\nabla\mathscr{L} \tag{S22}$$

This explains the rapid convergence observed in our experiments (e.g., stabilizing within 5 epochs). The learned error kernel effectively "preconditions" the gradient, straightening the optimization trajectory in the high-dimensional phase space.

## Supplementary note 4: Parameter $\beta$ and the Asynchronous Parallel Update Mechanism

### 4.1 The Role of Parameter $\beta$

As delineated in the main text, the target optical field for each layer is constructed
as:

$$\widehat{U_m^{l_m}} = U_m^{l_m} - F(\beta E_{m+1}^{l_{m+1}}) \otimes \delta_{m+1}^{local} \tag{S23}$$

Here, the parameter $\beta$ functions as a coefficient that scales the amplitude of the corrective term derived from the subsequent layer. Specifically:

A larger $\beta$ value signifies a stronger demand for the current layer ($m$) to alter its output to substantially aid the subsequent layer ($m$+1) in reducing its local target error $\left(\delta_{m+1}^{local}\right)$.

A smaller $\beta$ value results in a gentler corrective signal, leading to more modest adjustments in the preceding layer's target output.

Crucially, the local target error $\delta_{m+1}^{\text{local}}$ at layer $m$+1 is itself driven by errors propagated from even deeper layers, ultimately originating from the global loss at the network's output. Thus, $\beta$ modulates the intensity of this back-propagating corrective signal without altering its fundamental direction. Our experimental finding that the final accuracy is insensitive to the specific value of $\beta$ (provided $\beta > 0$) confirms that the update direction remains valid, while the value of $\beta$ effectively modulates the step size, primarily influencing the convergence rate.

4.2 Principles of Asynchronous Parallel Update

The formulation in Eq. (S1) inspires a fundamental departure from the synchronous update paradigm of standard backpropagation. It enables an asynchronous and parallel update strategy for the B-ONN, which operates as follows:
Triggered Forward Pass and Local Error Calculation: When the optical field propagates to layer $m$+1 and its output $U_{m+1}^{J_{m+1}}$ is obtained, the local target error $\delta_{m+1}^{local}=U_{m+1}^{J_{m+1}}-\widehat{U_{m+1}^{J_{m+1}}}$ can be immediately computed, where $\widehat{U_{m+1}^{J_{m+1}}}$ was set in the previous iteration.

Immediate Error Forwarding and Target Update: This locally compute $\delta_{m+1}^{local}=U_{m+1}^{J_{m+1}}-\widehat{U_{m+1}^{J_{m+1}}}$ is immediately used to compute a new target output $\widehat{U_m^{J_m}}=U_m^{J_m}-F(\beta E_{m+1}^{J_{m+1}})\otimes\delta_{m+1}^{local}$ for the preceding layer ($m$) via Eq. (S1). This step does not require waiting for the forward pass to complete through the entire network.

Parallel Weight Optimization: Upon receiving its new target $\widehat{U_m^{J_m}}$, layer $m$ can immediately commence its own weight optimization process (e.g., adjusting the phase profile of its diffractive elements). Critically, this local optimization at layer $m$ proceeds concurrently with the ongoing forward propagation of the light field to former layers ($m$+1, $m$+2, ...) and their subsequent computations.

This process creates a computationally efficient "pipeline". While the first training sample triggers the update of layer $m$, the optical signal from the second sample might be propagating through layer $m+1$, and so on. The gradient can descend correctly because the target for each layer is guided by a corrective signal that, through the chain of local errors, ultimately aligns with the global optimization objective.

4.3 Advantages of the Asynchronous Update Scheme

This asynchronous parallel configuration offers significant advantages over the conventional synchronous backpropagation scheme, particularly for physical implementations.

Greatly Reduced Training Latency and High Throughput: The scheme eliminates

the "global delay" inherent in synchronous backpropagation, where every layer must wait for the error to backpropagate from the output. The training speed is no longer linearly constrained by the total depth of the network. Instead, the system's throughput is primarily determined by the processing time of the slowest individual layer, analogous to a high-efficiency assembly line.

Superior Hardware Friendliness: The architecture is physically intuitive. It only requires establishing direct communication links between adjacent optical layers (e.g., using photodetectors and modulators) to pass local error signals, thereby avoiding the need for a complex central controller to distribute gradients. Furthermore, it drastically reduces the memory footprint since there is no need to store the intermediate results of all layers simultaneously for a full backward pass.

True Physical Parallelism: Different physical layers can work simultaneously in different stages. Some process new forward passes while others concurrently optimize their weights based on newly received targets. This fully exploits the innate parallel processing potential of optical computing.

Enhanced System Robustness: The relative independence of layer-wise updates grants the system greater resilience. A temporary delay or fault in one layer does not necessarily halt the entire training process, as other layers can continue their respective computations and updates.

In summary, the asynchronous update mechanism, governed by the scalable corrective term in Eq. (S20), is not merely an algorithmic alternative but a transformative feature tailored for physical neural networks. It effectively decomposes the global optimization problem into localized, concurrent sub-tasks, enabling low-latency, hardware-efficient, and robust on-chip learning that aligns perfectly with the properties of optical systems.

## Supplementary Note 5: Optical Gradient Computation Units

### 5.1 Optical Complex-Amplitude Encoding Unit (OCEU)

The error field at the ($k$+1)-th layer is generated using a double-phase holography method implemented on a phase-only spatial light modulator (SLM). This approach encodes the complex error field $Ae^{i\varphi}$ (containing amplitude $A$ and phase φ) into a phase-only hologram, overcoming the SLM's inherent limitation in directly modulating complex amplitude. Based on Euler's formula $Ae^{i\varphi}=(e^{i\alpha}+e^{i\beta})/2$ , the complex field is decomposed into two phase-only components, where $\alpha=\varphi+\arccos(A)$ and $\beta=\varphi-\arccos(A)$, with amplitude $A$ normalized to [0,1]. The resulting phase maps $\alpha(x,y)$ and $\beta(x,y)$ are encoded in a checkerboard pattern on the SLM according to pixel parity. A 4f imaging system with a spatial low-pass filter in the Fourier plane selects the +1 diffraction order to physically reconstruct the complex error field, ensuring wavefront coherence by using the same laser source as in forward propagation.

### 5.2 Optical Local Error Unit (OLEU)

For the $m$-th layer ($m\neq k+1$), the error field is computed using a Mach–Zehnder

interferometer. The optical fields $U_m^{l_m}$ and $\widehat{U_m^{l_m}}$ are combined at a 50:50 beam splitter, whose Jones matrix yields outputs $E_{out2} \propto i(U_m^{l_m}+\widehat{U_m^{l_m}})$. To achieve subtraction, a piezoelectric actuator introduces a $\pi$-phase shift in the $\widehat{U_m^{l_m}}$ path via active feedback locking, transforming the output to $E_{out2} \propto i(U_m^{l_m}-\widehat{U_m^{l_m}})$. The resulting field $\delta_m^{local}$ is modulated by the error matrix $E_m^{l_m}$ loaded on an SLM, producing the error sensitivity $d_{m\text{-}1}=E_m^{l_m} \otimes \delta_m^{local}$, which replaces explicit derivative calculation. The subsequent target field $\widehat{U_{m\text{-}1}^{l_{m\text{-}1}}}$ is computed optically using a similar interferometric subtraction scheme, where $F$ denotes Fresnel propagation and scaling factor $\beta$ is controlled via an optical attenuator.

5.3 Optical Parallel Gradient Unit (OPGU)

The phase gradient $\Delta\phi_m=2Re\{iF(P_m)\cdot U_m^{l_m}(\text{-}r_m)\odot \delta_m^{local}\}$ is computed via spatial interferometry. The fields $F(P_m)\cdot U_m^{l_m}$ and $\delta_m^{local}$ are interfered at a beam splitter after introducing a $\pi/2$ phase delay in one path (equivalent to multiplication by $i$). The interference intensity $I(x,y)=|A|^2+|B|^2+2Re(A\cdot iB)$ contains the target term alongside background intensities. Phase-shifting interferometry with multiple phase-stepped measurements extracts the pure $2Re(A\cdot iB)$ term. The error matrix gradient $\Delta\theta_m$ is set proportional to $\Delta\phi_m$, enabling simultaneous phase updates for both the weight matrix $R_m^{l_m}$ and error matrix $E_m^{l_m}$.

## Supplementary note 6: TensorFlow simulations and Detailed Training Configuration

We conduct two types of simulations in this work. For the chip-integrated T-ONN and B-ONN, which are physically fabricated, we employ the angular spectrum method to simulate near-field light propagation1, while also accounting for the material's refractive index. For the SLM-modulated network, we adopt Fresnel diffraction to model the convolutional operations and align with far-field propagation conditions2. The simulations are implemented using the TensorFlow (version 2.9.0, Google Inc.) framework under Python 3.83. To ensure reproducibility, the specific network architecture and training hyperparameters are detailed below:

Network Architecture: The network comprises 3 optical layers, two convolutional layers followed by one fully connected layer. The number of kernels in

each layer is designed to scale with task complexity. The first convolutional layer utilizes $N$ kernels (generating $N$ feature maps), and the second layer expands to $N \times N$ kernels (generating $N \times N$ feature maps), where $N$ is a scalable hyperparameter determined by the classification requirements. In our implementation, for the 10 class classification task we set $N$=9, resulting in 9 kernels in the first layer and 81 kernels in the second layer; for a simpler 4- class task, $N$=4 (i.e., 16 kernels in the second layer) would suffice. This scalable design allows the architecture to be adapted flexibly to different problem scales and optical hardware constraints. No digital normalization or nonlinear activation functions are applied, preserving a purely linear optical model throughout the forward propagation.

Initialization: The trainable forward phase parameters (ϕ) are initialized to zeros (representing a planar wavefront) to ensure unbiased initial signal transmission. The backward error kernels are initialized with random noise uniformly distributed between 0 and $2pi$.

Optimization Hyperparameters: The cross-entropy loss is used as the objective function. The Adam optimizer is applied for parameter updates with a learning rate of 0.007, a batch size of 32, and a training duration of 20 epochs. B-ONN Specific Parameters: The feedback scaling factor is set to $\beta$=0.9, and the error phase update rate is set to $\gamma$=0.1 .

**Supplementary note 7: Theoretical Analysis of Phase Smoothness**

7.1 High-Frequency Artifacts in Standard Backpropagation

Standard backpropagation in diffractive optical networks computes gradients via the chain rule through coherent wave propagation. In such systems, the optical field is often dominated by high-frequency interference patterns (speckle noise). Consequently, the calculated gradient, denoted as $g_{bp}$, inherently captures these high-spatial-frequency fluctuations. Furthermore, because these mathematically derived high-frequency gradients are structurally correlated across different training instances to minimize the empirical loss, they constructively accumulate over mini-batch updates rather than averaging out. When optimizing non-convex loss landscapes using first-order methods like SGD, the training process tends to follow these noisy gradient signals into sharp, fragile local minima. This results in phase masks that overfit to specific pixel-level interference artifacts rather than learning robust, generalized optical features.

7.2 Smoothness Induction Mechanism in B-ONN

In contrast to standard backpropagation, the B-ONN framework introduces a physics-constrained learning that naturally induces smoothness in the learned phase masks. Mathematically, the training process can be formulated as a joint optimization problem for the forward parameters Rand the backward error propagation parameters

$E$:

$$\min_{E,R} L(O,T) \quad subject\ to\ g_{local}(E,R) \approx g_{bp}(R) \tag{S24}$$

This formulation enforces smoothness through two coupled mechanisms:

Diffraction as a Physical Low-Pass Filter: The local gradient update $g_{local}$ is derived from an optical error field propagated through the learned kernel $E$. Since$E$is implemented as a physical diffractive element with band-limited modulation capabilities, it cannot support arbitrary high-frequency noise. The physics of diffraction (governed by the limited numerical aperture and propagation distance) acts as a natural spatial low-pass filter, suppressing high-frequency components during the error propagation process.

Implicit Regularization via Gradient Alignment: The constraint $g_{local} \approx g_{bp}$ requires the learned kernel $E$ to approximate the true descent direction. While the random initialization of backward kernels may introduce high-frequency speckles into the initial local error fields, these specific components are stochastically independent of the input data. Over iterative mini-batch updates, these instance-specific, zero-mean fluctuations undergo statistical cancellation. As a result, the optimization process naturally distills only the consistent, low-frequency structural updates, providing a robust, low-frequency update signal for the forward parameters $R$.

## Supplementary note 8: Nano Printing

The microstructures are fabricated using the Two-Photon Polymerization (TPP) technique, a high-precision laser direct writing process based on femtosecond laser pulses and non-linear two-photon absorption. The system employs a femtosecond fiber laser (Coherent Fidelity II) with a central wavelength of 780 nm, generating pulses with a duration of 55 fs at a repetition rate of 70 MHz. The laser beam is guided through a series of optical components, including an acousto-optic modulator for precise power control, and is focused into the photoresist using a high-numerical-aperture oil immersion objective (e.g., 63x/1.4 NA or 25x/0.8 NA). Following the printing process, the sample undergoes a development step to remove the non-polymerized resin. This typically involves rinsing in a solvent such as isopropanol (purity > 99.5%) for 20-30 minutes, followed by gentle drying at room temperature.

## Supplementary note 9: Quantitative Analysis of Computational Overheads

### 9.1 Analysis of Floating-Point Operation Complexity

In traditional networks, convolution operations are considered intensive matrix multiplication operations. The training process involves three stages: forward inference, error backpropagation, and weight gradient computation, all relying on the energy-inefficient floating-point multiply-accumulate (MAC) instructions.

In contrast, B-ONN architecture use the principle of light to complete complex spatial domain transformations at the speed of light. The computational primitives are downgraded from high-power sliding window multiplication and addition to low-power pixel subtraction.

For a network with $L$ layers, an input resolution of $I \times I$, a kernel size of $K \times K$, and $C$ channels, the floating-point computation cost per sample per training iteration is defined as follows:

$$FLOPs_{T-ONN} \approx 3 \times L \times (2 \cdot I^2 \cdot C \cdot K^2) \quad (S25)$$

Where the coefficient 3 represents the entire training loop (forward, error backpropagation, gradient update). The term $K^2$ represents the computational overhead of the sliding window in digital computation: to compute a pixel in the output image, the multiplier must multiply and add points within the $K \times K$ region one by one, and its complexity increases quadratically with the receptive field area.

$$FLOPs_{B-ONN} \approx L \times (2 \cdot I^2) \quad (S26)$$

In B-ONN, the convolution operation is completed by the overall evolution of the light field in space, and its physical process smooths out the complexity difference caused by the kernel size ($K$). The electrical overhead comes only from the complex field reconstruction formula in the four-step interferometry:

$$E_{recon} \propto (I_1 - I_3) + j(I_4 - I_2) \quad (S27)$$

For $I \times I$ pixels, reconstructing each layer of the complex field only requires two subtraction operations between the real and imaginary parts, and its computational cost is strictly dependent on the image resolution $I$, and is independent of the kernel size $K$.

## 9.2 Memory Access and Data Traffic

In the von Neumann architecture, the energy consumption of data movement is typically 2-3 orders of magnitude higher than pure arithmetic operations. The total dynamic memory traffic involved in a single weight update is defined as $T$:

$$T_{T-ONN} \approx L \times I^2 \times C \times Precision \ (Bytes) \quad (S28)$$

$$T_{B-ONN} \approx 1 \times I^2 \times C \times Precision \ (Bytes) \quad (S29)$$

T-ONN must follow global gradient dependency, caching the entire forward activation value $a^{(l)}$ of each layer into SRAM/DRAM until backpropagation is complete. B-ONN, on the other hand, introduces a calculate and discard mechanism, where data is physically consumed during spatial propagation, eliminating the need to maintain a deep data stack at the digital end.

## 9.3 Analysis of Training Latency

Training latency is a key metric for measuring the real-time adaptation and online learning capabilities of edge AI. In T-ONN, weight updates can only be performed after the complete forward and backward chains are completed. This causes latency to accumulate linearly with network depth $L$ , accompanied by severe pipeline stalls.

B-ONN allows forward inference and Optical Parallel Gradient Unit (OPGUs) to operate quasi-synchronously at the physical level. For B-ONN, the bottleneck of training speed shifts from computational path depth to the physical refresh rate of the spatial light modulator (SLM). Currently, though the use of digital micromirror devices (DMDs) can significantly improve the weight update rate, the physical refresh limit determined by the modulator hardware response remains the core bottleneck restricting B-ONN from becoming an ultra-high-speed real-time learning system.

## Supplementary note 10: Hardware Overheads

### 10.1 Spatial Bandwidth Product (SBP)

In physical optics computing architectures, the Spatial Bandwidth Product (SBP) is a core metric for measuring system complexity. The B-ONN architecture employs a space-for-memory trade-off, avoiding the need for high-power, high-speed digital buffers by increasing the static masking area. In other words, the parallelism of computation is directly mapped to the physical space occupied.

For a system with $L$ layers, $k$ kernels per layer, and $I \times I$ resolution, the total pixel requirement $P_{total}$ is defined as:

$$P_{total} = \gamma \cdot (P_{fwd} + P_{err}) \cdot (1+\beta) \tag{S30}$$

where $\gamma$ represents the redundancy factor required for optical complex amplitude coding (OCEU) through pure phase holography; $P_{fwd} = L \cdot k \cdot I^2$ represents the number of pixels in the forward path; $P_{err}$ represents the number of pixels in the paired auxiliary error layers; and $\beta$ represents the ratio of additional space required for diffraction guard bands to alignment tolerance.

For a an optical neural network with 9 layers, 9 kernels and 112*112 resolution

$$P_{fwd} + P_{err} \approx 2 \times 10^6 pixels \tag{S31}$$

After accounting for $\gamma \approx 2.5$ and $\beta \approx 0.5$, the actual footprint expands to approximately $7.5 \times 10^6$ pixels. B-ONN achieves complete decoupling of electrical storage bandwidth requirements during training through approximately 3 times spatial redundancy.

### 10.2 Fabrication and Alignment Complexity

In the integrated chip path, maintaining wavelength-level feature size consistency is extremely challenging due to the limited processing precision of two-photon polymerization (TPP). Furthermore, with increasing layer count, the extremely compact three-dimensional layout not only induces residual stress deformation but also poses a risk of physical collapse to deep suspended structures due to the inherent mechanical instability of the materials and capillary forces during development. Currently, supercritical $CO_2$ drying or the design of support scaffolds have proven to be effective solutions for enhancing the mechanical stability of such

structures . In the spatial optical path, due to the cascading of multiple independent OPGU and OLEU modules, the expansion of system size increases the physical space required. Meanwhile, when attempting to increase the spatial bandwidth product by reducing pixel size, the enhanced edge diffraction effect induces crosstalk, leading to a degradation in phase coherence. To overcome the limitations of traditional diffraction elements in subwavelength scale manipulation, utilizing the superior wavefront manipulation capabilities of metasurfaces to replace traditional diffraction layers has become an inevitable evolutionary direction for improving system fidelity and compact integration.

## Supplementary Figures

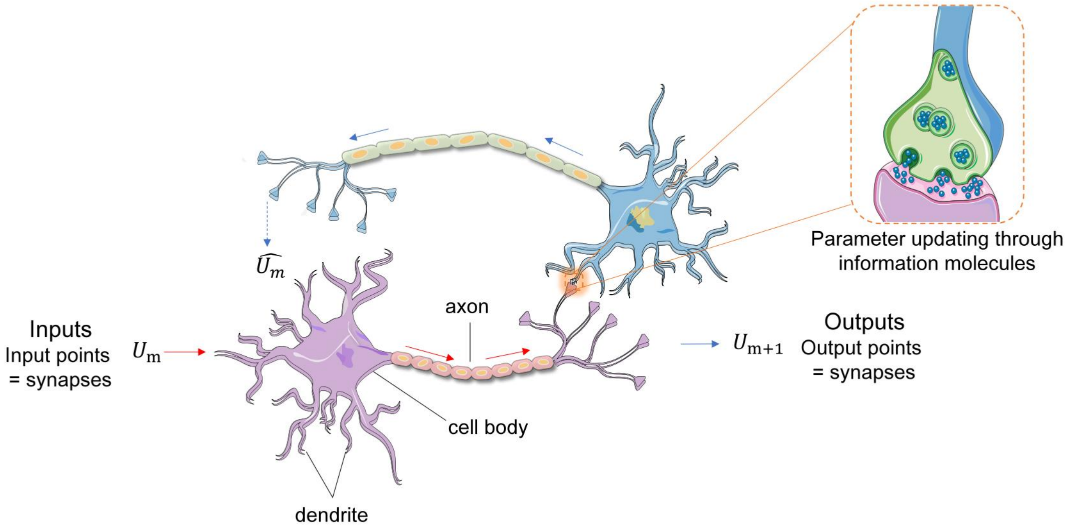


**Figure. S1** Schematic of local synaptic plasticity in biological neural circuits. The strength of a synaptic connection (highlighted in orange) is modified based on the co-activity of the pre- and post-synaptic neurons, without requiring a global error signal. This process is driven by localized "messenger molecules".

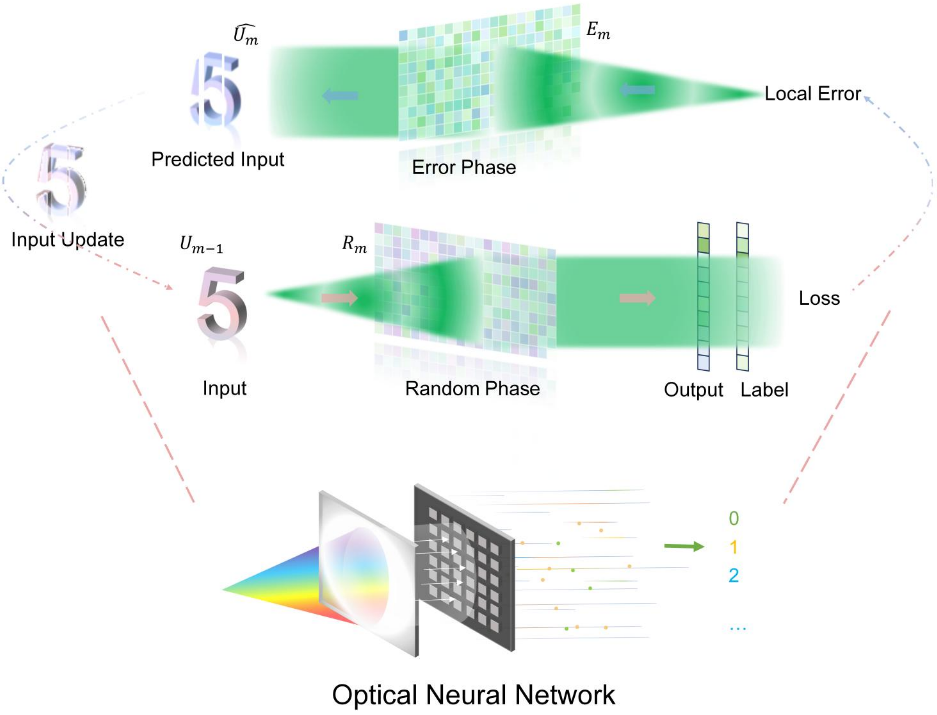


**Figure. S2** This schematic illustrates a backpropagation-free learning process for classifying an input digit. The output field ($U_m$), generated from the input after propagation through a diffractive layer ($R_m$), is compared to a target label to compute a loss. A local error signal, combined with a random phase for exploration, is fed back to update the input, forming a closed-loop, brain-inspired learning rule. This local

update mechanism enables efficient online training without global error backpropagation.

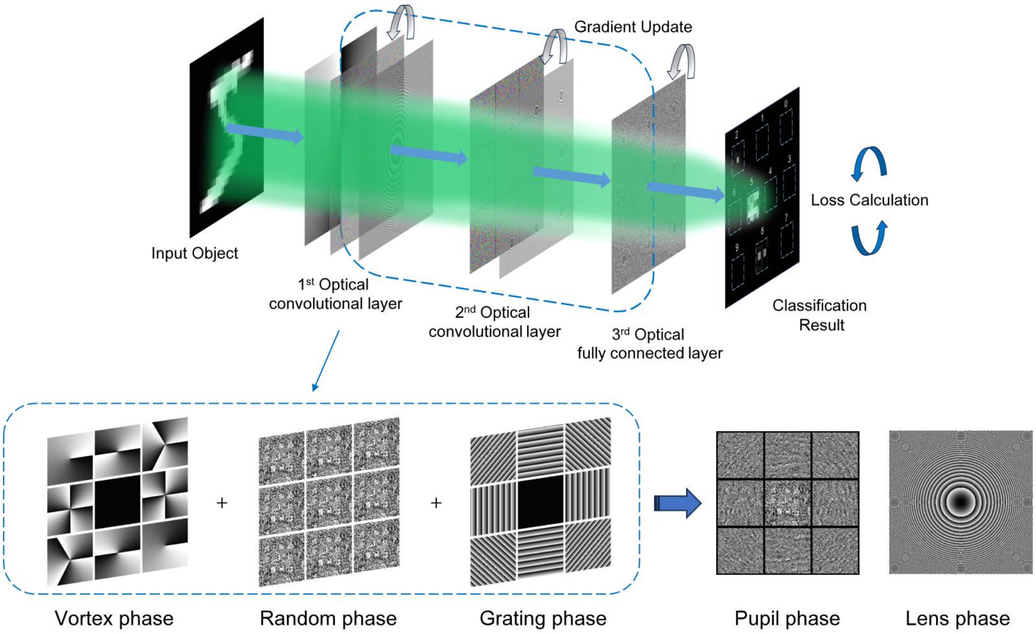


**Figure. S3** The diagram outlines the shared optical pathway for information processing in both the broadcast and trainable optical neural networks. An input object is sequentially transformed by a series of optical layers: two convolutional layers followed by a fully connected layer. The inset details the composition of a kernel in the first convolutional layer, constructed by synthesizing three functional phase components: a vortex phase (providing structural priors for edge extraction), a random phase (serving as trainable weights), and a grating phase (acting as a spatial router for beam steering). The final output is used for classification, with the resulting loss guiding the gradient-based update of the network's phase parameters. Green arrows indicate the direction of the optical data flow.

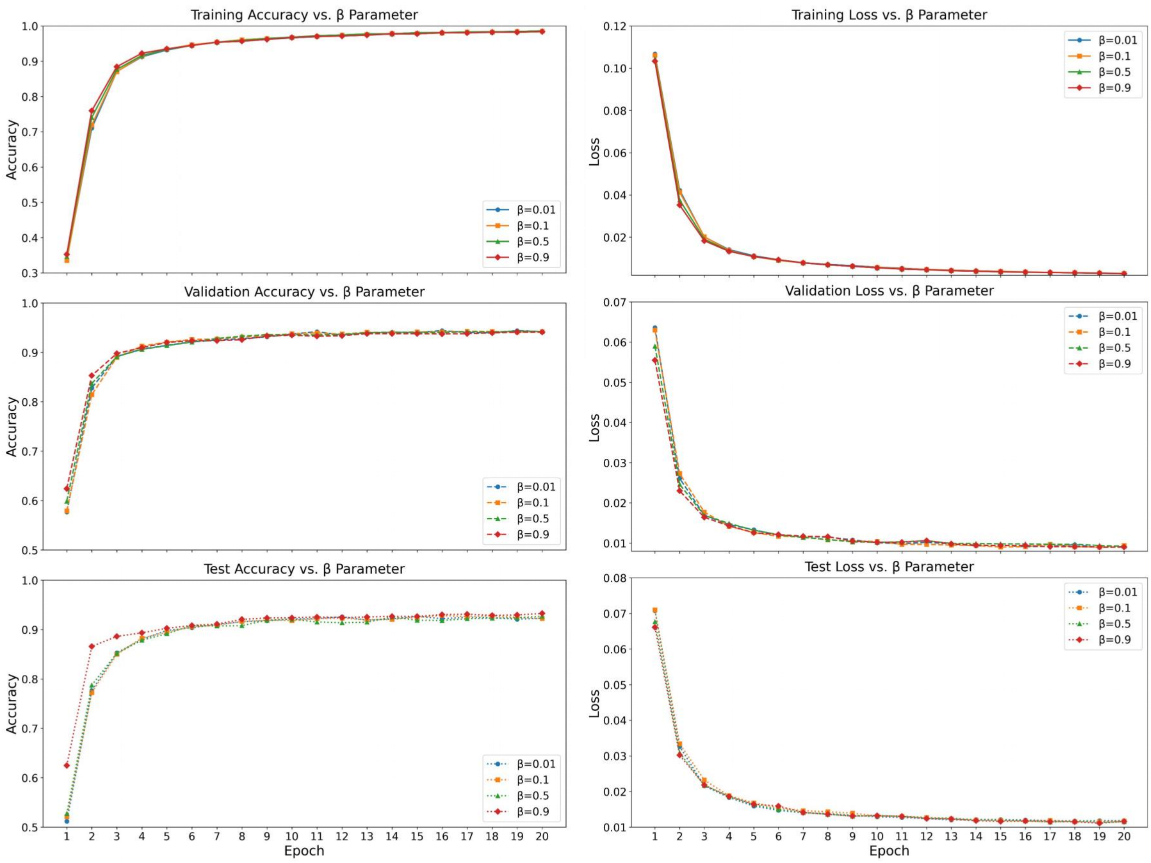


**Figure. S4** The performance (accuracy and loss) on the training, validation, and test sets across 20 training epochs is shown for different values of the feedback scaling factor $\beta$ (0.01, 0.1, 0.5, 0.9). The top, middle, and bottom rows correspond to the training, validation, and test sets, respectively.

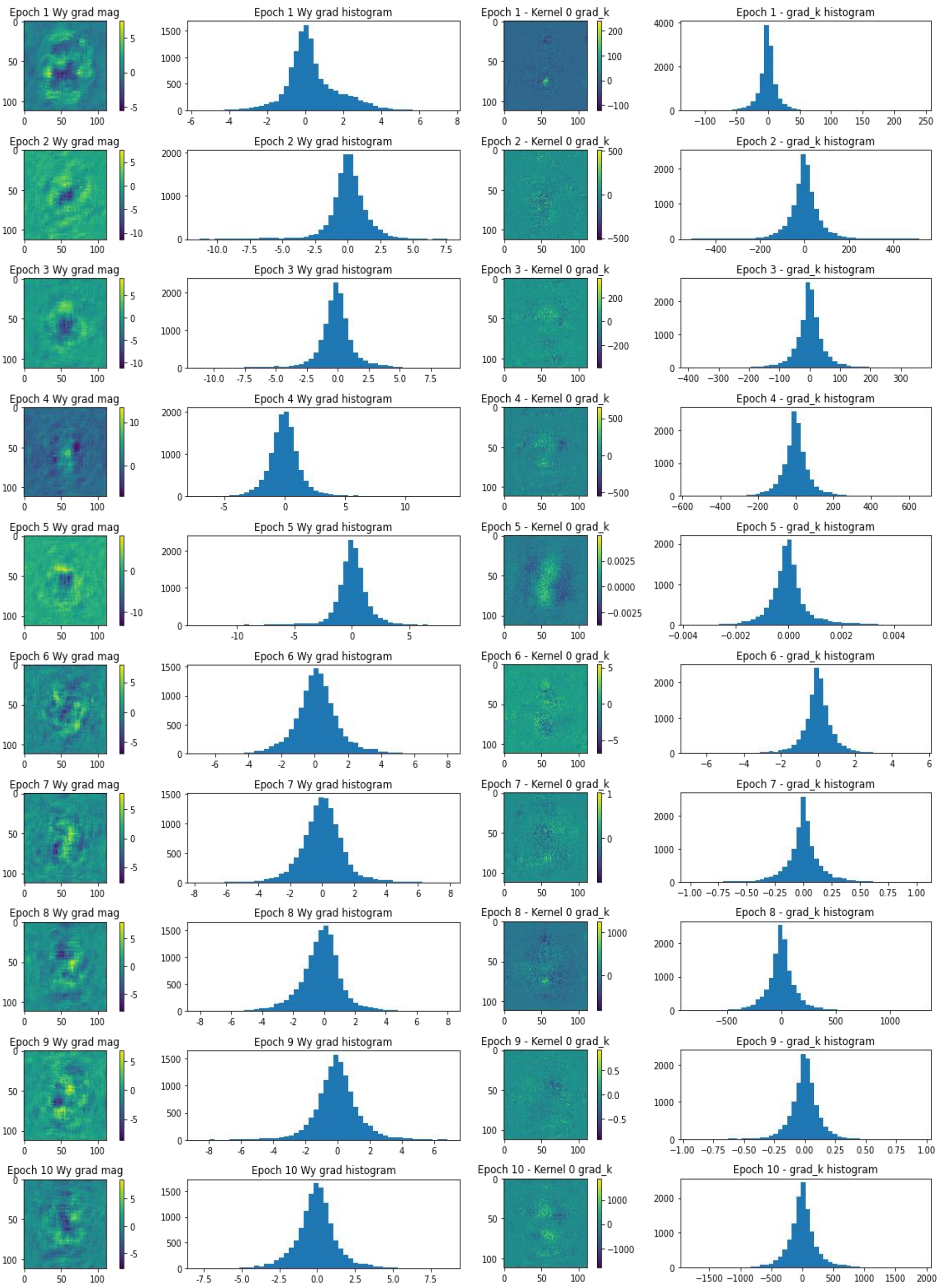
Epoch 1 Wy grad mag
Epoch 1 Wy grad histogram
Epoch 1 - Kernel 0 grad_k
Epoch 1 - grad_k histogram
Epoch 2 Wy grad mag
Epoch 2 Wy grad histogram
Epoch 2 - Kernel 0 grad_k
Epoch 2 - grad_k histogram
Epoch 3 Wy grad mag
Epoch 3 Wy grad histogram
Epoch 3 - Kernel 0 grad_k
Epoch 3 - grad_k histogram
Epoch 4 Wy grad mag
Epoch 4 Wy grad histogram
Epoch 4 - Kernel 0 grad_k
Epoch 4 - grad_k histogram
Epoch 5 Wy grad mag
Epoch 5 Wy grad histogram
Epoch 5 - Kernel 0 grad_k
Epoch 5 - grad_k histogram
Epoch 6 Wy grad mag
Epoch 6 Wy grad histogram
Epoch 6 - Kernel 0 grad_k
Epoch 6 - grad_k histogram
Epoch 7 Wy grad mag
Epoch 7 Wy grad histogram
Epoch 7 - Kernel 0 grad_k
Epoch 7 - grad_k histogram
Epoch 8 Wy grad mag
Epoch 8 Wy grad histogram
Epoch 8 - Kernel 0 grad_k
Epoch 8 - grad_k histogram
Epoch 9 Wy grad mag
Epoch 9 Wy grad histogram
Epoch 9 - Kernel 0 grad_k
Epoch 9 - grad_k histogram
Epoch 10 Wy grad mag
Epoch 10 Wy grad histogram
Epoch 10 - Kernel 0 grad_k
Epoch 10 - grad_k histogram

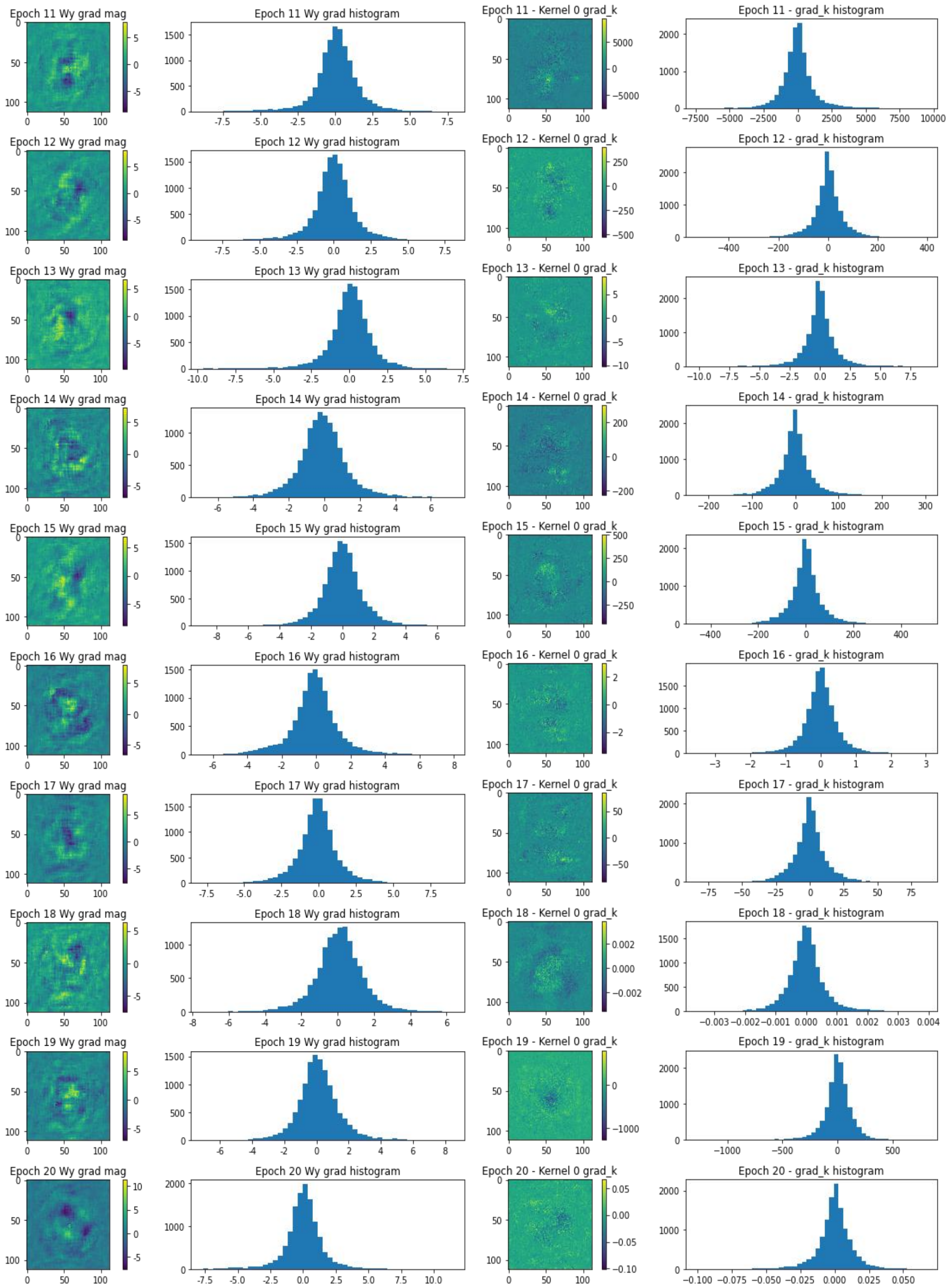


**Figure. S5** Temporal evolution of stochastic phase gradients in B-ONN and T-ONN (Epochs 1–20). The first column displays the spatial distribution maps of the kernel gradients, while the second column illustrates the corresponding gradient histograms of B-ONN. Correspondingly, the third and fourth column depicts the gradient magnitude maps and histograms of T-ONN.

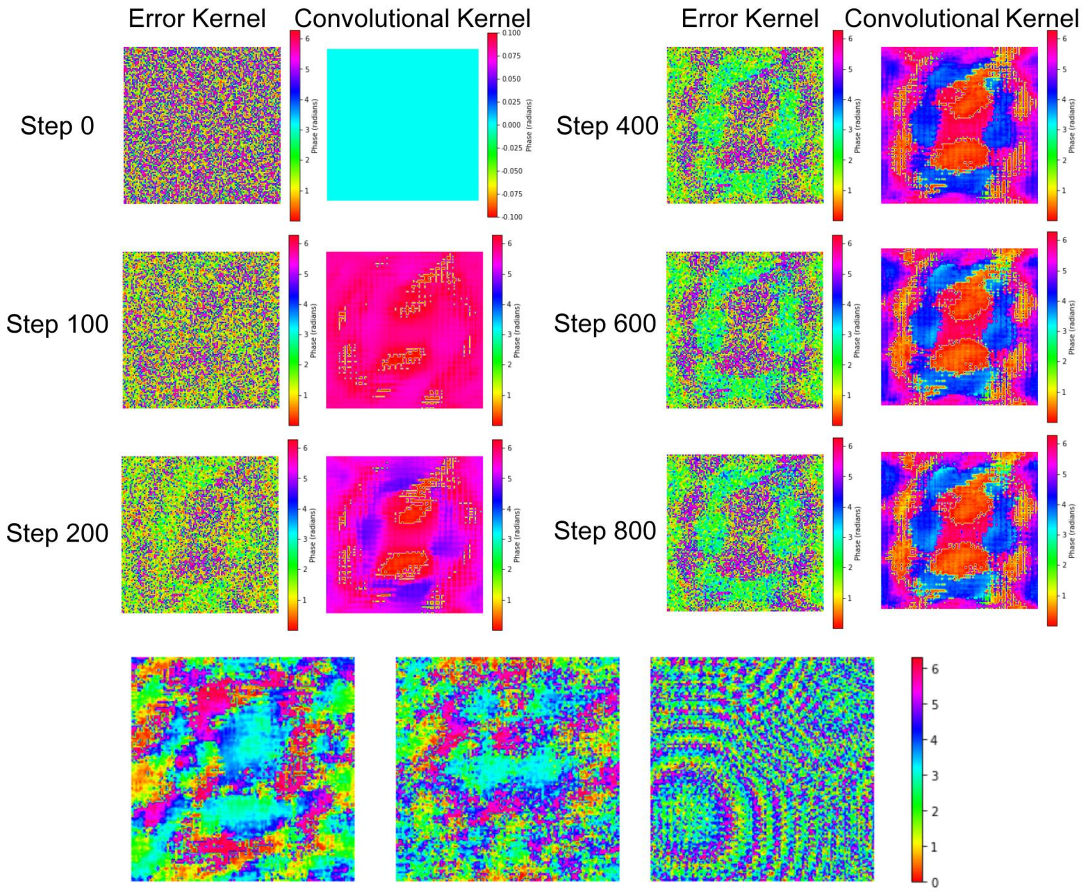


**Figure. S6** Evolution of spatial phase distributions for Error and Convolutional Kernels across training steps 0–800. The color scale indicates phase values in radians. At Step 0, the Convolutional Kernel shows a uniform phase profile while the Error Kernel exhibits random distribution; subsequent steps display non-uniform spatial textures in both kernels.

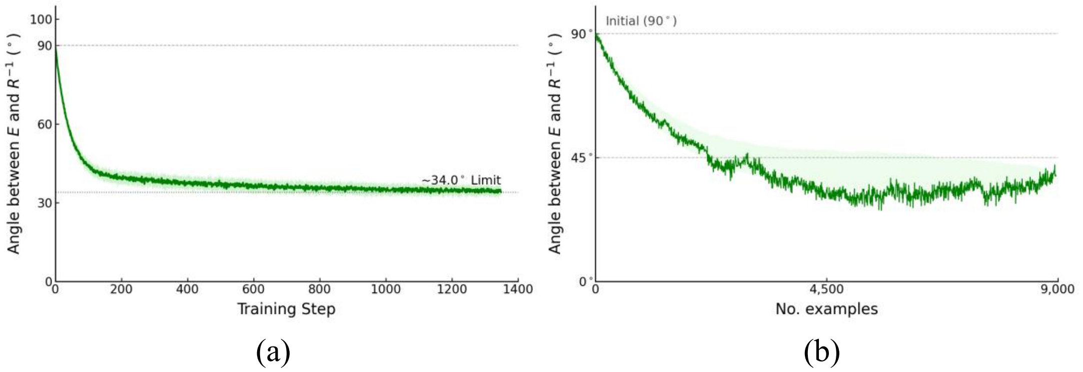


(a) (b)

**Figure. S7** Evolution of the angle between $E$ and $R^{-1}$ during training. (a) The angular trajectory plotted against training steps, saturates at a limit of approximately 34°. (b) The angular trajectory plotted against the number of training examples. The process initiates at an orthogonal state (90°) and stabilizes around 45°.

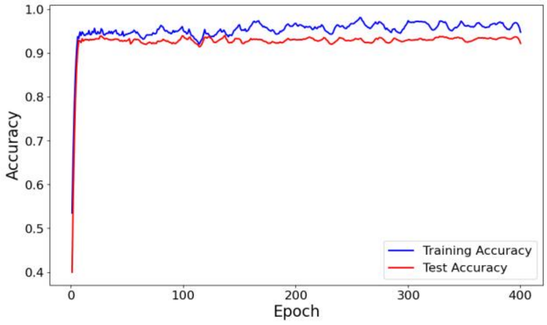


(a)

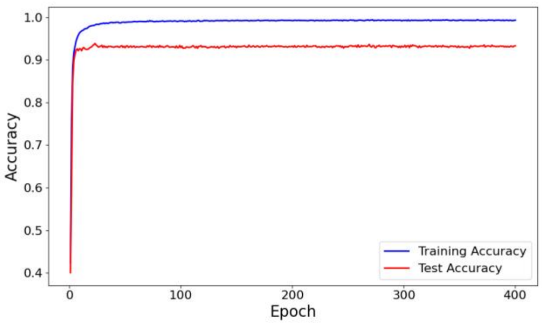


(b)

**Figure. S8** Long-term convergence stability and performance limits over 400 epochs. (a) Training and test accuracy of the traditional backpropagation-based optical neural network (T-ONN) on the MNIST dataset. (b) Training and test accuracy of the proposed B-ONN architecture under identical conditions.

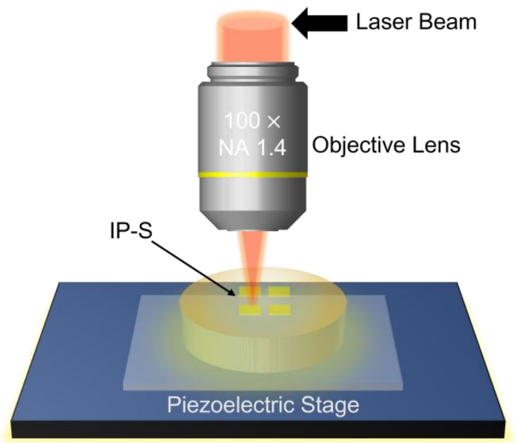


**Figure. S9** A femtosecond laser beam is tightly focused by a high-numerical-aperture (NA 1.4) 100x oil-immersion objective lens onto the surface of a photoresist (IP-S), which is spin-coated on a substrate. The piezoelectric nano-positioning stage moves the substrate with nanometric precision in three dimensions relative to the fixed laser focus. At the focal point, the high peak intensity of the pulsed laser triggers two-photon absorption within the photoresist, initiating a localized polymerization reaction and solidifying the IP-S resin.

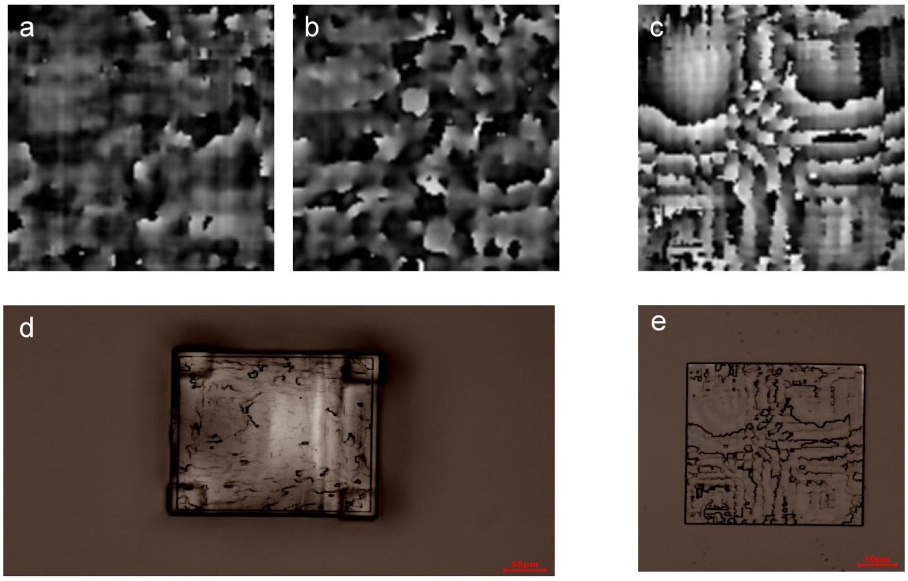


**Figure. S10** (a, b) Phase maps of the first and second layers in the chip-integrated dual-layer B-ONN, (c) Phase profile of the single-layer B-ONN implementation, (d, e) Optical micrographs (20×) of the nano printed B-ONN chip.

## References


1. Lillicrap, T. P., Cownden, D., Tweed, D. B. & Akerman, C. J. Random synaptic feedback weights support error backpropagation for deep learning. *Nat Commun* **7**, 13276 (2016).
2. Zee, T., Ororbia, A. G., Mali, A. & Nwogu, I. A Robust Backpropagation-Free Framework for Images. Preprint at http://arxiv.org/abs/2206.01820 (2023).
3. Lin, X. et al., All-optical machine learning using diffractive deep neural networks. Science 361, 1004–1008 (2018).
4. Nanoscribe. https://www.nanoscribe.com/en/.
5. Zhang, Y. *et al.* Memory-less scattering imaging with ultrafast optical convolutional neural networks.
6. Zhu, H., Chen, Y., Hu, G. & Yu, S. Contrastive Learning via Local Activity. Electronics 12, 147 (2022).
7. Shi, J. et al. Anti-noise diffractive neural network for constructing an intelligent imaging detector array. Opt. Express 28, 37686 (2020).
8. Goi, E., Schoenhardt, S. & Gu, M. Direct retrieval of Zernike-based pupil functions using integrated diffractive deep neural networks. Nat Commun 13, 7531 (2022).
